# Spliceosomal Sm core assembly: AlphaFold 3 predicted structure and phosphorylation-dependent regulation of the human 6S complex

**DOI:** 10.1016/j.csbj.2025.12.013

**Published:** 2025-12-16

**Authors:** Matthias Grimmler, Marco Reinhart, Sebastian Alers, Christoph Peter

**Affiliations:** a Institute for Biomolecular Research, Hochschule Fresenius, University of Applied Sciences, Idstein, Germany; b DiaServe Laboratories GmbH, Iffeldorf, Germany; c GfA GmbH, Pfronten, Germany; d DiaSys Diagnostic Systems GmbH, Holzheim, Germany; e Institute of Molecular Medicine I, Medical Faculty, Heinrich Heine University Düsseldorf, Düsseldorf, Germany

**Keywords:** Spliceosome, U snRNP assembly, CLNS1A, ULK1, AlphaFold, Protein structure prediction

## Abstract

The *in vivo* assembly of uridine-rich small nuclear ribonucleoproteins (U snRNPs), the central catalytic components of the spliceosome, is a highly organised, multi-step process orchestrated by several multi-protein complexes. Structural analyses have provided valuable insights into their overall architecture. However, critical information on the regulation of U snRNP assembly is still lacking. In this study, we used AlphaFold 3 to model the human 6S intermediate complex consisting of full-length pICln and five Sm proteins SmD1/D2/E/F/G. The available crystal structure used truncated non-vertebrate proteins, omitting the highly flexible C-terminal segments to permit crystallisation. However, the C-terminus of pICln has since been recognised as regulatory region, making full-length computational models an appropriate way to elucidate its structural and functional roles. By integrating modelling with biochemical data from previous studies, our results support a model in which the phosphorylation-dependent regulation of the pICln–SmG interface facilitates downstream assembly steps in vertebrates, including the regulated displacement of pICln by the SmD3/B dimer. According to this model, ULK1-dependent serine phosphorylation in the C-terminal α-helix of pICln may abrogate the secondary structure and weakens its interaction with SmG, favouring ring opening. This complementary *in silico* approach elucidates the roles of regulatory regions in pICln that were previously inaccessible to crystallographic analysis and provides a framework for targeted experimental validation.

## Graphical Abstract

**Figure:**
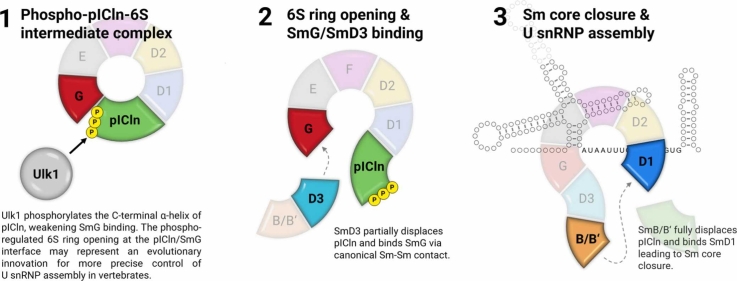

## 1 Introduction

Pre-mRNA splicing is catalysed by the spliceosome, a large ribonucleoprotein complex in which uridine-rich small nuclear ribonucleoproteins (U snRNPs) provide splice-site recognition and, together with U snRNAs, form the RNA-based catalytic core [1], [2]. A key step in U snRNP biogenesis is the formation of a heptameric ring, the Sm core, comprising seven Sm proteins (SmB, D1, D2, D3, E, F, and G) [3], [4]. Although Sm proteins and U snRNAs assemble spontaneously into functional U snRNPs *in vitro*, their formation *in vivo* is channelled through a multi-step, chaperone-assisted pathway [5], [6], [7], [8]. The chaperone pICln participates in two assembly intermediates: the pICln–D3/B complex and the ring-shaped hetero-hexameric 6S complex containing pICln and SmD1/D2/E/F/G. In both intermediates, pICln prevents premature or incorrect assembly steps and pre-organises the Sm proteins into the order required for the mature heptameric Sm core [9], [10], [11]. Notably, pICln is essential for U snRNP assembly even in unicellular fission yeast [12]. The SMN complex, comprising SMN, Gemin2–8 and Unrip, then receives the Sm proteins from both the 6S and pICln–D3/B intermediate and assembles them onto the snRNA [7], [9], [10], [13], [14], [15].

The precise mechanism by which pICln is displaced from the SmD1/D2/E/F/G pentamer remains unresolved. *In vitro* reconstitution studies underscore the role of the highly conserved SMN complex in this step, demonstrating that the recombinant SMN complex can simultaneously accept Sm proteins and dissociate pICln [9], [16], [17]. The only experimental 6S structure was reported by Grimm et al. (2013), using a chimeric complex comprising a truncated pICln protein from *Drosophila melanogaster* lacking the C-terminal region [10]. The authors identified the pICln/SmG contact, between β0 of pICln and β7 of SmG, as the smallest and most divergent within the 6S complex, suggesting that this represents a “predetermined breaking point” [10].

Recent work established an additional regulatory layer in vertebrates. As demonstrated by Schmitz et al. (2021), the serine/threonine kinase Unc-51-like kinase 1 (ULK1) selectively weakens the pICln–SmG interaction by phosphorylating Ser193, Ser195 and Ser197 in the C-terminus of human pICln [18]. ULK1-dependent phosphorylation of pICln induces a conformational transition of the 6S complex from a compact, closed ring to a more elongated, open state, as shown by analytical ultracentrifugation [18]. Esser et al. (2023) subsequently showed that ULK1-dependent phosphorylation increases the efficiency of U1 snRNP assembly by promoting Sm protein transfer to the SMN complex [19]. In ULK1 knockout cells, the transfer of Sm protein onto the U snRNA is strongly reduced, indicating that ULK1 enhances Sm transfer in vertebrates [19].

In this study, we used AlphaFold 3 as a complementary approach to model the human 6S complex and to analyse the potential consequences of C-terminal phosphorylation of pICln. AlphaFold has significantly transformed the field of structural biology [20]. It uses deep learning algorithms trained on large structural databases to predict protein folding, domain interactions, and binding interfaces [20]. The first AlphaFold version addressed the challenge of predicting a protein's tertiary structure from its amino acid sequence, achieving near-experimental accuracy for many well-folded domains, whereas intrinsically disordered or highly flexible regions are predicted with low confidence, because these regions do not adopt a single stable conformation in solution [20]. AlphaFold 2 introduced an end-to-end deep learning approach to further improve prediction accuracy [20], [21]. AlphaFold 3 (AF3) further improves on its predecessors by modelling interactions with other molecules, and incorporating information on post-translational modifications, such as phosphorylation [22]. AlphaFold 3 is a diffusion-based, generative model that directly predicts the atomic coordinates of multimolecular complexes, including chemically modified residues [22]. AF3 additionally reports confidence measures, including a per-residue predicted Local Distance Difference Test score (pLDDTs; 0–100), where values < 50 indicate very low and > 90 very high local confidence, and a Predicted Aligned Error (PAE), reflecting confidence in the relative placement of domains or chains [22].

Previous structural studies of the 6S complex have relied on *in vitro* reconstitution using truncated pICln proteins from non-vertebrate species [10]. This approach was necessary due to the poor expressibility and crystallisability of the full-length human pICln, particularly its regulatory C-terminus [10]. As the resulting experimental structure inherently excludes any insight into the vertebrate-specific ULK1-mediated regulation, deep learning-based modelling provides a feasible method to investigate the full-length human 6S complex. By integrating computational modelling with biochemical insights from our previous studies [18], [19], this work offers testable hypotheses for future experimental validation.

## 2 Material and methods

### 2.1 Protein sequences and structure prediction

The amino acid sequences of the human proteins included in this study were retrieved from UniProtKB (Table 1) or the NCBI protein database (Table 2). The x-ray diffraction structure of the chimeric 6S intermediate, comprising Sm proteins from *Mus musculus* and *Xenopus laevis*, and a truncated form of *Drosophila melanogaster* pICln lacking amino acids 90–125 and carrying an H144A mutation (PDB ID: 4F7U), was obtained from the PDB database [10]. Structure predictions were performed using the AlphaFold 3 webserver (https://alphafoldserver.com), accessed between 10 February and 2 March 2025 [22]. Default parameters were applied, and five structural models were generated for each complex. The highest-ranked model based on the predicted local distance difference test (pLDDT) score was selected for subsequent analyses. To assess structural plausibility, the AF3-derived atomic coordinates (CIF files) were cross-checked by superposition with the experimental 6S complex using PyMOL’s *super* command. Positional deviation was quantified using the root mean square deviation (RMSD), which measures the average distance between corresponding atoms after optimal superposition. RMSD values were visualised using a modified ColorByRMSD PyMOL script (https://pymolwiki.org/index.php/ColorByRMSD), adapted to colour the RSMD value from blue (minimum) to yellow (maximum). The effect of serine phosphorylation was modelled using the integrated post-translational modification option of the AlphaFold 3 webserver, which allows the direct specification of phosphorylated residues prior to structure generation. The phosphorylated and non-phosphorylated models were compared for conformational differences and alterations in inter- and intra-protein interactions.

**Table 1:** Protein sequences used for structure prediction of the Sm core and 6S complex.

UniProt ID	Length	Protein [Homo sapiens]	Sm core	6S complex	Colour (HEX)
P54105	237 aa	Methylosome subunit pICln	-	+	Green (#00ff00)
P14678–1	240 aa	Small nuclear ribonucleoprotein B’	+	-	Orange (#ff8000)
P62318	126 aa	Small nuclear ribonucleoprotein D3	+	-	Cyan (#00ffff)
P62314	119 aa	Small nuclear ribonucleoprotein D1	+	+	Blue (#0080ff)
P62316	118 aa	Small nuclear ribonucleoprotein D2	+	+	Yellow (#ffff00)
P62304	92 aa	Small nuclear ribonucleoprotein E	+	+	Grey (#e6e6e6)
P62306	86 aa	Small nuclear ribonucleoprotein F	+	+	Violet (#ff80ff)
P62308	76 aa	Small nuclear ribonucleoprotein G	+	+	Red (#ff0000)

**Table 2:** Protein sequences used for evolutionary conservation analysis of pICln.

NCBI ID	Length	Species	Name	Class (Order)
NP_001284.1	237 aa	*Homo sapiens*	Human	Mammalia (Primates)
NP_076160.1	241 aa	*Mus musculus*	House mouse	Mammalia (Rodentia)
NP_001003288.1	235 aa	*Canis lupus fam.*	Dog	Mammalia (Carnivora)
XP_046765352.1	238 aa	*Gallus gallus*	Chicken	Aves
XP_060627213.2	241 aa	*Anolis sagrei*	Brown anole	Reptilia
NP_001081766.1	241 aa	*Xenopus laevis*	African clawed frog	Amphibia
XP_006006363.1	243 aa	*Latimeria chalumnae*	Coelacanth	Lobe-finned fish
NP_571499.2	249 aa	*Danio rerio*	Zebrafish	Ray-finned fish
XP_071798056.1	231 aa	*Asterias amurensis*	Sea star	Asteroidea
XP_002129420.1	221 aa	*Ciona intestinalis*	Sea squirt	Ascidiacea
NP_611237.2	215 aa	*Drosophila melanogaster*	Fruit fly	Insecta

### 2.2 Structural visualisation and analysis

Molecular visualisation and structural analysis were conducted using PyMOL (Schrödinger, LLC; version 2.5.0). Secondary structure elements were assigned from the AF3-derived atomic coordinates using PyMOL’s built-in dss algorithm, which infers secondary structure based on both backbone geometry and hydrogen-bonding patterns. All assignments were cross-checked with the DSSP algorithm. Notably, DSSP annotated the characteristically curved β-strands of the Sm proteins as single continuous elements (in line with existing PDB entries), while the dss algorithm assigned short internal loop interruptions. Because this more closely reflected the AF3-predicted backbone geometry, dss was used for all structural representations. To maintain compatibility with the canonical description of the five-stranded Sm fold (β1–β5), the internal β-strand numbering was harmonised with the DSSP-based convention. Further details are provided in Supplementary Figure S16. The ribbon representation of the protein complexes was coloured by subunit identity (Table 1) or based on the pLDDTs of the central backbone carbon (Cα). Model confidence was categorised and coloured as follows: very high (pLDDTs >90; blue, #004CCA), high (90 > pLDDTs >70; cyan, #49C4EE), low (70 > pLDDTs >50; yellow, #FFD539), very low (pLDDTs <50; red, #FF7143). Side chains and backbone groups at the modelled protein–protein interfaces, geometrically compatible with hydrogen-bonding and hydrophobic contacts, were annotated with PyMOL and the Arpeggio web server (http://bleoberis.bioc.cam.ac.uk/arpeggioweb) [23]. For the sake of clarity, unstructured internal loops or terminal regions (pLDDTs <70) that do not participate in the complex formation or overlap with high-confidence areas of interest have been hidden in the structural representations. The full-length protein complexes are depicted in Supplementary Figures S3 and S4 and Supplementary Videos S1 and S2.

Supplementary material related to this article can be found online at doi:10.1016/j.jallcom.2022.164017.

The following is the Supplementary material related to this article Video S1.

Video S1

Supplementary material related to this article can be found online at doi:10.1016/j.jallcom.2022.164017.

The following is the Supplementary material related to this article Video S2.

Video S2

### 2.3 Evolutionary conservation analysis

The evolutionary conservation of the pICln C-terminus was assessed using Clustal Omega multiple sequence alignment (Version 1.2.4; https://www.ebi.ac.uk/jdispatcher/msa/clustalo). The NCBI Reference Sequence of human pICln (NP_001284.1) was aligned against seven vertebrate homologues ranging from mammals (*Mus musculus, Canis lupus*) to fish (*Latimeria chalumnae, Danio rerio*) using default settings (Table 2). Residue-wise conservation was quantified using the online conservation scoring tool available at https://compbio.cs.princeton.edu/conservation/score.html [24]. Multiple sequence alignments in FASTA format were analysed using the Shannon entropy scoring method with a window size of 3 and BLOSUM62 substitution matrix, resulting in normalised scores ranging from 0 (low conservation) to 1 (strong conservation) per residue. Evolutionary analysis was extended to non-vertebrate species *Ciona intestinalis* (Chordata), *Asterias amurensis* (Deuterostoma) and *Drosophila melanogaster* (Protostoma) (Table 2).

## 3 Results

### 3.1 The pICln C-terminus is modelled to partially adopt an α-helical conformation

Previously, we demonstrated that ULK1-dependent phosphorylation of Ser193, Ser195, and Ser197 in the human pICln C-terminus interferes with pICln/SmG interaction, favours 6S ring opening and promotes transfer of Sm proteins to the SMN complex [18], [19]. However, the structural details of this vertebrate-specific regulatory mechanism remained elusive, as the only available experimental 6S structure lacks the C-terminus of pICln, which is predicted as an intrinsically disordered region (IDR) by classical prediction tools [25]. However, based on the 13Cα-13Cβ secondary chemical shifts in nuclear magnetic resonance (NMR) spectroscopy, Schedlbauer and colleagues have previously suggested that the central region of the pICln C-terminus may be able to adopt a preferred α-helical organisation [25]. Consistent with these experimental results, we observed that the AlphaFold Monomer v2.0 pipeline (AlphaFold protein structure database entry AF-P54105-F1-v4) predicts two consecutive α-helices in the C-terminus of human pICln spanning Tyr170 to His176 and Ala179 to Gln194; modelled with high to very high confidence (pLDDTs >70; Supplementary Figure S1). To examine how AlphaFold predicts the structure of human pICln within the assembled 6S intermediate, we used the AlphaFold 3 webserver to model the quaternary structure from the full-length human protein sequences.

### 3.2 pICln is modelled as an integral part of the 6S complex connecting SmG and SmD1

To evaluate the performance of AF3, we first predicted the quaternary structure of the mature Sm core comprising the seven full-length human Sm proteins, SmB’, D1, D2, D3, E, F, and G. As shown in Supplementary Figure S2, AF3 accurately predicted the heptameric toroidal structure of the Sm core, and modelled the canonical Sm-Sm contacts, namely β-sheets formed by two antiparallel β-strands. The central Sm folds forming five-stranded antiparallel β-sheets were modelled with the highest confidence (pLDDTs >90), while the structure and position of the internal loops and flexible terminal extension were predicted with lower confidence (pLDDTs <70; Supplementary Figures S2 and S3).

**Fig. 1:**
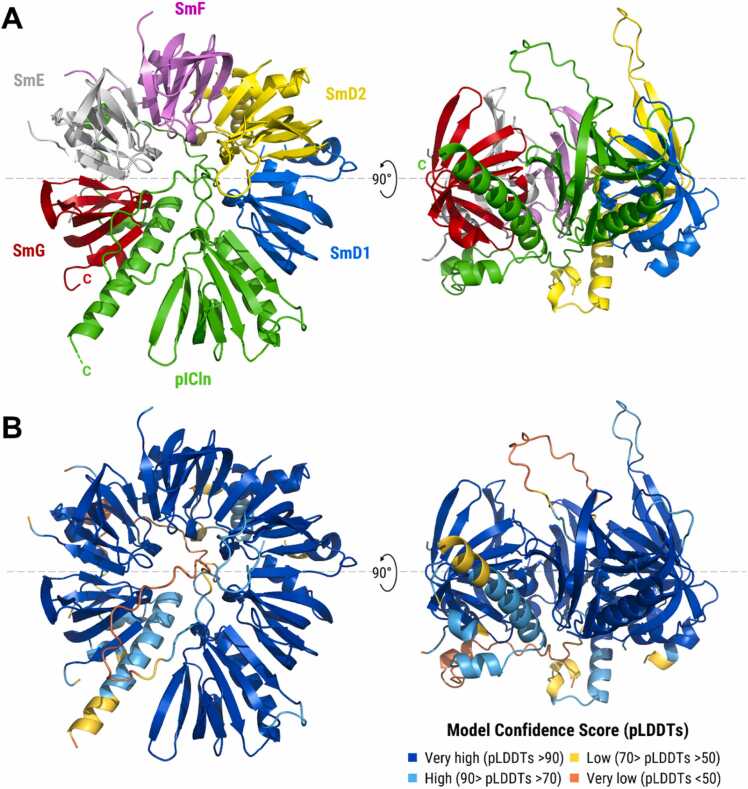
Structure of the human 6S complex predicted by AlphaFold 3 (Model 0). **(A)** Ribbon representation of the heterohexameric 6S intermediate comprising full-length human pICln and the five Sm proteins SmD1, D2, E, F, and G. The quaternary structure was predicted by AlphaFold 3 using the human reference sequences from UniProtKB (Table 1). **(B)** Ribbon representation coloured by pLDDT score ranging from very low (<50; red) to very high confidence (>90; blue).

Next, we predicted the quaternary structure of the human 6S complex comprising full-length pICln and the five Sm proteins SmD1, D2, E, F, and G (Fig. 1). In agreement with the experimental 6S structure, pICln was modelled as an integral part of the hexameric ring, interacting with SmG and SmD1 and serving as a placeholder for the SmD3/B dimer to be subsequently integrated (Fig. 1A). In line with Grimm et al [24], the β5-strand of pICln interacts with β4 of SmD1 to form an antiparallel β-sheet, resembling a canonical Sm-Sm contact. As seen for the mature Sm core, the central Sm folds of the five Sm proteins and the extended β-strand rich pleckstrin-homology (PH)-like domain of pICln (comprising β1-β7 and α1) were modelled with very high confidence (pLDDTs >90; Fig. 1B), while the unstructured internal regions of pICln (between β6 and β7 and between α1 and α2) were predicted with very low pLDDT score, consistent with their experimentally reported flexibility (Fig. 1B; Supplementary Figure S4). To visualise structural differences, we superimposed the AF3-predicted human 6S complex and the chimeric X-ray 6S structure (PDB 4F7U) in PyMOL and quantified positional deviations by root mean square deviation (RMSD) over aligned Cα atoms. The mean RMSD was 1.64 Å; RMSDs were consistently low across the Sm pentamer, suggesting a nearly identical core architecture, whereas significant deviations were limited to the pICln component (*Drosophila* pICln in the crystal versus human pICln in the AF3 model), affecting both the local structure and the positioning of pICln within the 6S assembly (Supplementary Figures S5 and S6).

**Fig. 2:**
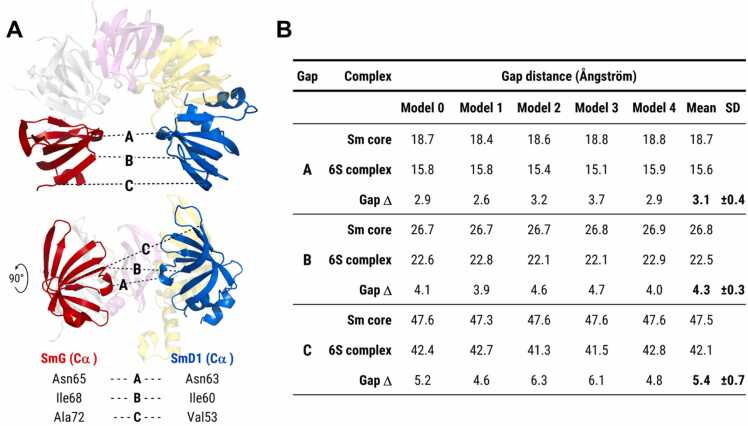
SmG–SmD1 gap distances in the AF3-modelled Sm core and 6S complex. **(A)** The distance between SmG and SmD1 was measured using PyMOL between three identical Cα positions in SmG (red) and SmD1 (blue). Gap A: SmG (Asn65) to SmD1 (Asn63); Gap B: SmG (Ile68) to SmD1 (Ile60); Gap C: SmG (Ala72) to SmD1 (Val53). **(B)** The gap distances in angstrom (Å) were determined for all five AF3 models (Model 0–4), and mean gap differences (Gap Δ) and standard deviations (SD) were calculated.

Next, we compared the distance between SmG and SmD1 in the AF3-predicted Sm core (gap occupied by SmD3/B) with the distance in the 6S complex (gap occupied by pICln). We measured the distances between the Cα atoms of three reference residues in SmG (Asn65, Ile68, Ala72) and SmD1 (Asn63, Val53, Ile60) using PyMOL for all five AF3 models (Fig. 2A). As shown in Fig. 2B, the mean SmG-SmD1 gap distance was larger in the heptameric Sm core compared to the hexameric 6S complex (ΔGap A: 3.1 ± 0.4 Å; ΔGap B: 4.3 ± 0.3 Å; ΔGap C: 5.4 ± 0.7 Å), suggesting that the AF3-modelled 6S complex represents a compact intermediate state that must undergo gap widening to enable the integration of the SmD3/B dimer.

### 3.3 The C-terminal α-helix of pICln is modelled to mediate the interaction with SmG

**Fig. 3:**
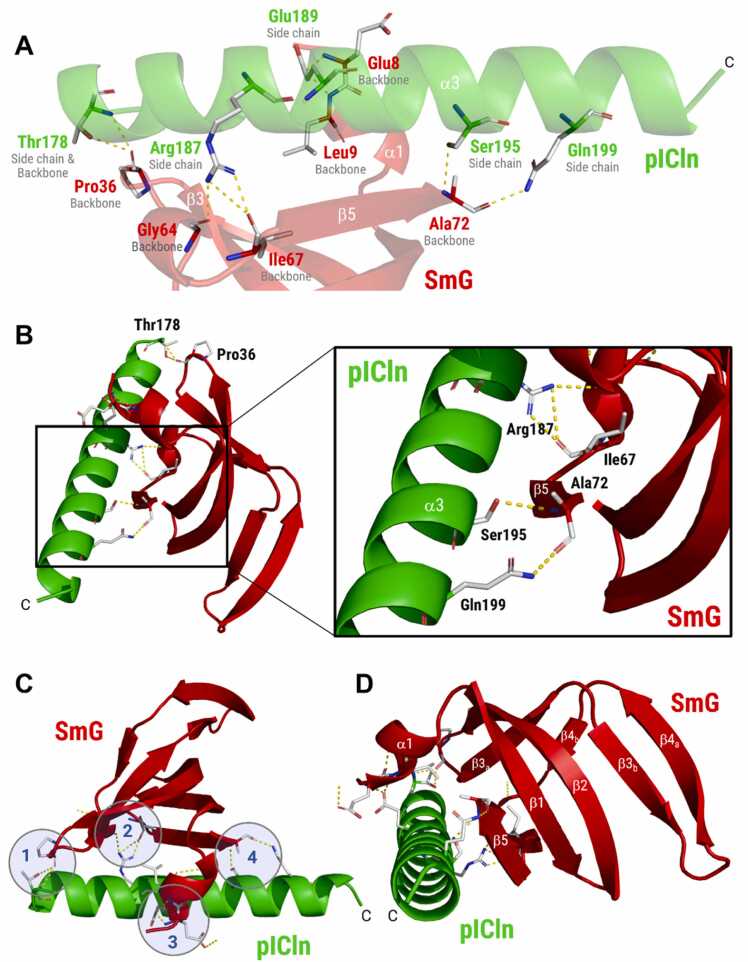
Detailed analysis of the pICln/SmG binding interface predicted by AlphaFold 3. **(A)** Ribbon representation of the entire C-terminal α-helix in pICln (green; α3) and the regions of SmG (red; α1, β3a and β5) modelled to mediate the pICln/SmG interaction (Model 0). Side chains of pICln and backbone atoms of SmG involved in hydrogen bonding (yellow dotted lines) are indicated. **(B)** Detailed view of the interaction between Arg187, Ser195, and Gln199 (located in pICln α3) and the backbone groups of SmG (located in β5). **(C)** Representation of the binding interface of SmG, mediating the interaction with pICln at four contact points (#1 to #4) along the α3-helix. **(D)** Alternative view of the pICln/SmG binding interface rotated by 90°. Position and succession of secondary structural elements in SmG are indicated, ranging from the N-terminal α-helix (α1) to the most C-terminal β-strand (β5).

Within the AF3-predicted 6S complex, the extended C-terminal α-helix of human pICln appears to mediate the contact between pICln and SmG (Fig. 1). The high local confidence between Thr178 and Ser197 (90 > pLDDTs >70), and the consistent placement of the helix across all five ranked AF3 models (Supplementary Figure S7) argued against a low-confidence artefact. Hence, we analysed the residues positioned to mediate the pICln/SmG interaction in more detail (Fig. 3).The AF3-modelled SmG binding residues of pICln include Arg187, Ser195, and Gln199, which are positioned to form hydrogen bonds with the backbone of SmG β5 at Ile67 and Ala72 (Fig. 3A/B). Thr178 forms a hydrogen bond with the SmG backbone at Pro36 (located in the β2-β3 loop), and Glu189 interacts via H-bonds with the SmG backbone at Glu8/Leu9 located in the short N-terminal α1-helix of SmG (Fig. 3A/B; Supplementary Video S3). In addition, several side chains lie within distance consistent with hydrophobic packing and weak aromatic interactions. Residues positioned to mediate such interactions include pICln Thr178, placed for a potential C–H···π interaction with SmG Phe37, and pICln Leu192, whose terminal methyl groups could form C–H···π contacts with the aromatic ring of SmG Phe12. SmG Met13 (α1) and pICln Tyr170 are positioned for additional S···π and C–H···π contacts (Supplementary Figure S8).

Supplementary material related to this article can be found online at doi:10.1016/j.jallcom.2022.164017.

The following is the Supplementary material related to this article Video S3.

Video S3

Overall, the SmG backbone is predicted to engage four contact regions along the pICln C-terminus, forming a binding interface that can be described as a multi-point “clamp” securing the extended α-helix of pICln (Fig. 3C/D). Three of these contact areas are modelled to line the helix from one side, while the N-terminal α1-helix of SmG acts as a “thumb” from the opposite side. The assigned secondary structure elements and pLDDT scores for pICln and SmG are summarised in Supplementary Figure S9.

### 3.4 pICln phosphorylation is modelled to interfere with α-helix formation and SmG binding

**Fig. 4:**
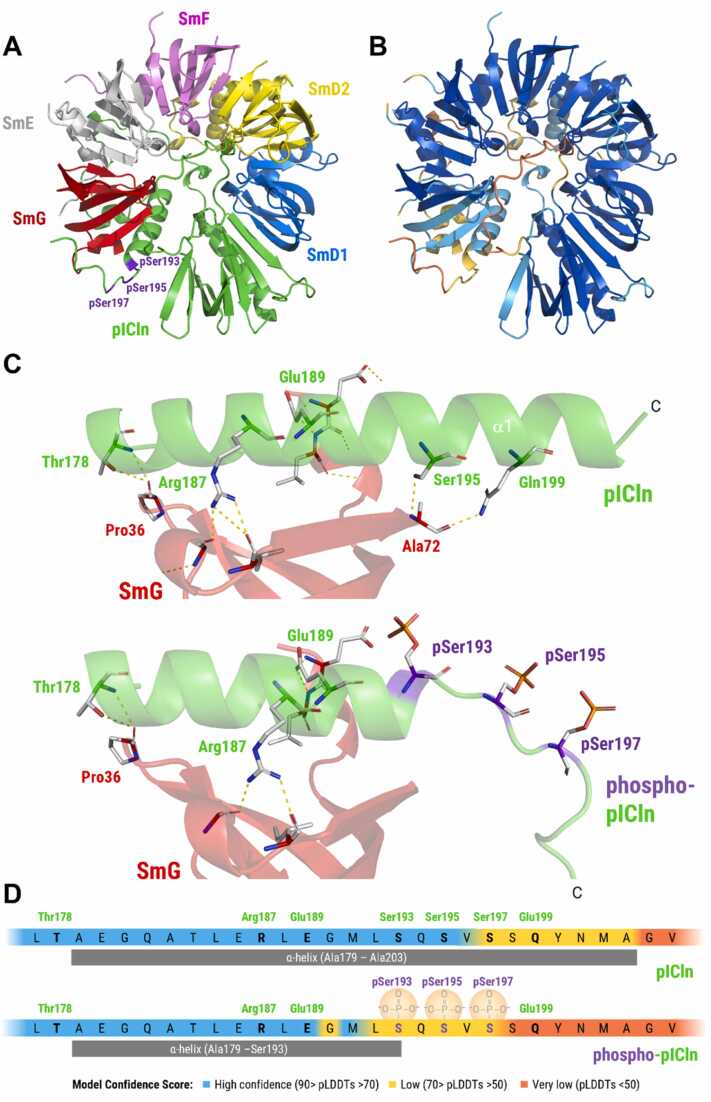
Structural alteration in the ULK1-phosphorylated 6S complex predicted by AlphaFold 3. **(A)** Ribbon representation of the 6S complex comprising human pICln phosphorylated at Ser193, Ser195, and Ser197 (Model 0). **(B)** Ribbon representation coloured by pLDDT score. **(C)** Close-up of the modelled structural changes upon phosphorylation. AF3 predicts local perturbation of the C-terminal α-helix, consistent with charge-induced unfolding, and side-chain orientations incompatible with hydrogen bonding **(D)** Summary of the modelled differences between the phosphorylated and non-phosphorylated state.

Ser195, one of three ULK1 phosphorylation sites in the pICln α3-helix, appears to represent a central binding residue at the predicted pICln/SmG binding site. Therefore, we tested whether the addition of negative charges could potentially interfere with the pICln/SmG interaction and modelled the entire 6S complex with human pICln phosphorylated at Ser193, Ser195, and Ser197 using the integrated post-translational modification option of the AF3 webserver. As shown in Fig. 4A/B, AF3 predicts local helical destabilisation consistent with potential charge-induced unfolding, resulting in a shorter α-helix (ranging to Ser193 instead of Met202) without significantly affecting the overall quaternary structure or the SmG-SmD1 gap distance (Supplementary Figure S10; Supplementary Video S4). Serine phosphorylation is predicted to disrupt the pICln/SmG interface at contact point #4, so that Ser195 and Gln199 lie beyond contact distance to the SmG backbone, thereby abolishing the hydrophobic contacts modelled in the unphosphorylated state (Fig. 4C; Supplementary Figure S11). The assigned secondary structure and pLDDT scores for the phosphorylated and non-phosphorylated C-terminus are summarised in Fig. 4D.

Supplementary material related to this article can be found online at doi:10.1016/j.jallcom.2022.164017.

The following is the Supplementary material related to this article Video S4.

Video S4

### 3.5 SmG binding residues in pICln are highly conserved among vertebrates

**Fig. 5:**
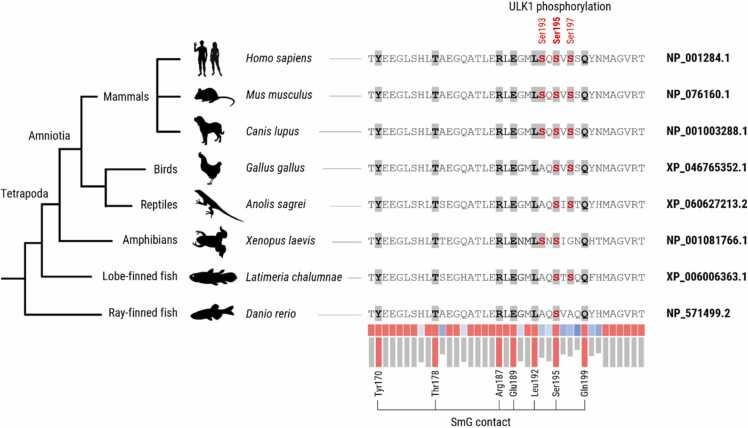
Evolutionary conservation of the modelled SmG-binding residues in pICln among vertebrates. Conservation of pICln residues (i) positioned to mediate SmG contact (Tyr170, Thr178, Arg187, Glu189, Leu192, Ser195, Gln199) and/or (ii) reported as ULK1 phosphorylation sites (Ser195, Ser197, Ser199) was assessed by Clustal Omega multiple sequence alignment using the NCBI reference sequence of human pICln and seven vertebrate orthologues (Table 2). Residue-wise conservation was quantified using the Shannon entropy scoring method, yielding normalised scores between 0 (low conservation; blue→white) and 1 (full conservation; red).

We identified seven pICln side chains along the C-terminal α-helix that are positioned to mediate binding to SmG. We therefore assessed the evolutionary conservation of these putative contact residues (Fig. 5). All seven amino acids (Tyr170, Thr178, Arg187, Glu189, Leu192, Ser195 and Gln199) are fully conserved among vertebrates, from ray-finned fish (*Danio rerio*) to humans (*Homo sapiens*). At the modelled SmG interface, the contacts are main-chain (carbonyl/amide) rather than side-chain dependent, while the aromatic SmG residues are conserved from vertebrates to *Drosophila* (data not shown). Notably, the pICln C-terminus shows a gain of serine residues over vertebrate evolution: Ray-finned fish contain only the central serine corresponding to human Ser195, whereas lobe-finned fish, amphibians, reptiles and birds have acquired an additional serine (either Ser193 or Ser197). A third adjacent serine emerged during the evolution of eutherian mammals, giving rise to the tri-serine ULK1 phosphorylation cluster (Ser193, Ser195 and Ser197) described by Schmitz et al. and Esser et al [18], [19].

For extended evolutionary analysis, three additional non-vertebrate sequences were included, representing successive outgroups within the Bilateria: *Ciona intestinalis* (Tunicate; distant chordate), *Asterias amurensis* (Echinoderm; distant deuterostome) and *Drosophila melanogaster* (Insecta; protostome). Notably, the pICln C-terminus differs markedly in both length and primary sequence, with very low local sequence similarity between vertebrates and the non-vertebrate outgroups (Supplementary Figure S12). This suggests that the pICln C-terminus acquired a defined functional role specifically within the vertebrate lineage, potentially reflecting a lineage-specific refinement and an additional regulatory layer that is largely absent in non-vertebrate ancestors.

## 4 Discussion

In this study, we combine previously established experimental evidence for ULK1-enhanced U snRNP biogenesis in vertebrates [18], [19] with deep learning-based modelling to propose a mechanistic framework for this regulatory mechanism. AlphaFold 3 predicts an extended C-terminal α-helix in human pICln, with high confidence (90 > pLDDT >70) between Thr178 and Ser195, that is placed at the pICln/SmG contact within the human 6S complex, offering a structural hypothesis for how ULK1-mediated phosphorylation in the C-terminus of human pICln is able to (i) strongly reduce its affinity for SmG, (ii) induce a more open 6S conformation, and (iii) increase the transfer efficiency of Sm proteins to the SMN complex [18], [19]. Based on this AF3 model, we hypothesise that the phosphorylation-induced modulation of the pICln/SmG interface may enhance the initial steps of 6S ring opening and favour pICln displacement, which can be facilitated by the SMN complex alone *in vitro*, thereby creating favourable conditions for subsequent U snRNP assembly steps in vertebrate cells.

Our results are conceptually consistent with the work of Grimm and colleagues, who performed extensive biochemical and structural analyses of the 6S intermediate [10]. However, their crystal structure (PDB 4F7U) was solved using a chimeric complex comprising a C-terminally truncated *Drosophila* pICln ortholog, because the full-length human pICln and Sm proteins could not be stably expressed and crystallised [10], which prompted us to model the complete human 6S complex using AlphaFold 3. Grimm et al. reported that the pICln/SmD1 interface resembles a canonical Sm-Sm contact comprising an antiparallel β-sheet, consistent with our findings [10]. In contrast, the authors identified the pICln/SmG contact as the smallest and most divergent within the 6S complex [10]. More precisely, in the x-ray diffraction structure of the 6S intermediate, the C-terminally truncated *Drosophila* pICln engages SmG through a short β-strand (β0; residues 1–6), which forms a parallel β-sheet with β5 of SmG [10]. In contrast, using the full-length human pICln sequence, AF3 does not reproduce this β-sheet pairing with SmG β5. Instead, the C-terminal α-helix of pICln is placed at the pICln/SmG interface, with side chains arranged for hydrogen-bonding and hydrophobic packing against SmG.

Notably, phosphorylation of pICln by ULK1 favours 6S ring opening and increases U snRNP assembly efficiency in vertebrates [18], but is not a prerequisite for pICln dissociation from the 6S intermediate. Since the core SMN assembly machinery is able to displace pICln from the SmD1/D2/F/E/G pentamer *in vitro*, it is reasonable to assume that pICln-release is facilitated by the evolutionarily conserved SMN complex. Chari et al. (2008) demonstrated that recombinant human SMN complexes can accept Sm proteins while simultaneously displacing pICln [9], and Hu et al. (2023) showed that efficient pICln release requires a minimal five-membered SMN complex (SMN, Gemin2, Gemin6–8) in fission yeast [16]. Hence, in these *in vitro* systems, pICln is removed in the complete absence of C-terminal phosphorylation. In addition, since pICln remained bound in the crystallised 8S complex, which also contains Gemin2/SMN, Grimm et al. concluded that additional conformational changes, possibly mediated by the C-terminus of SMN or other subunits of the SMN complex, are required for complete pICln dissociation [10]. Furthermore, the SmD1–SmG gap opening is too narrow and must be widened by snRNA binding, indicating that further structural rearrangements are required after the release of pICln to enable subsequent SmD3/B incorporation [26]. Therefore, ULK1-mediated phosphorylation seems to provide an additional regulatory layer in vertebrates, which modulates the pICln/SmG contact, whereas the release of pICln from the 6S complex and the subsequent incorporation of the SmD3/B dimer are promoted by other mechanisms, including SMN-driven structural rearrangements and conformational changes induced by the snRNA [10], [26].

Phosphorylation of serine and threonine residues is a widespread post-translational modification that enables rapid, reversible, and dynamic regulation of protein-protein interactions by fine-tuning binding specificity and/or affinity [27]. Depending on the exact position and context, the addition of negative charges can trigger conformational changes that either enhance or inhibit protein binding [27]. One of the best-studied examples is the α-helix-mediated interaction between p53 and MDM2, which is extensively regulated by serine/threonine phosphorylation [28]. Phosphorylation introduces a negative charge that disrupts the helical binding interface of p53 and reduces its affinity for MDM2 [29]. Based on the AF3 model presented in this study, it is tempting to propose a similar mechanism for the ULK1-mediated regulation of pICln/SmG interaction, although the lower pLDDT score and apparent loss of helicity could also reflect limited training coverage for phosphorylated residues, leading to lower model confidence for the modified sequence. Preliminary electrostatic surface potential (ESP) mapping (Supplementary Figure S13) indicates that phosphorylation at Ser193/Ser195/Ser197 would introduce a contiguous negative patch on the helix, which may reduce electrostatic complementarity to the facing SmG surface. As these findings are model-derived, they should be regarded as hypotheses pending experimental validation.

While X-ray crystallography and cryo-electron microscopy (cryo-EM) are both restricted to proteins that can be recombinantly expressed and purified in sufficient quality, deep learning-based structure prediction relies solely on amino acid reference sequences. However, its accuracy is highly dependent on the quality of the algorithm, the availability of homologous structural data, and the composition of the training dataset. In AlphaFold 2, the *Evoformer module* used multiple sequence alignments (MSAs) to infer structural features [21], [22]. In AlphaFold 3, this module is replaced with the *Pairformer module*, which focuses more directly on how different parts of a protein interact, enabling the prediction of regions with low sequence homology where reliable MSAs are not available [22]. Furthermore, while AlphaFold 2 used a *structure module* to predict protein structures by modelling torsion angles and the positions of side chains and backbones in a stepwise manner, AlphaFold 3 uses a deep learning-based *diffusion module*, directly predicting the positions of each atom in three-dimensional space [22]. Despite these improvements, AlphaFold 3 is not free of limitations. As a generative model, it can “hallucinate” secondary structure elements in intrinsically disordered regions. Abramson et al. (2024) reported that additional training on disordered proteins, combined with disorder annotations into the cross-distillation set, strongly reduced this tendency and markedly improved the model’s ability to distinguish structured from intrinsically disordered regions [22]. Nevertheless, hallucinated helices can still appear and are typically accompanied by very low pLDDT values (<50) [22], [30]. However, in the presented AF3 models, the C-terminal α-helix of pICln exhibits high local confidence (90 > pLDDTs >70) from Thr178 to Ser195. The helix is consistently placed at the pICln/SmG interface across all five AF3 models (Supplementary Figure S7), and AF3 assigns intermediate confidence to its relative placement against SmG (mean PAE: 8.3 ± 0.17 Å; Supplementary Figure S14). In addition, the non-generative AlphaFold 2 model (v2.3.2) independently recapitulates the C-terminal α-helix in pICln and the pICln/SmG interface (Supplementary Figure S15), which collectively argues against a low-confidence hallucination of the generative AF3 model. Finally, the discrepancy with the chimeric X-ray structure may arise from alternative conformations of the 6S intermediate, with our AlphaFold models capturing a transient C-terminal engagement instead of the β0-mediated β-sheet seen crystallographically.

Together, the published biochemical and cell biological data indicate that the core mechanism of pICln-release from the 6S intermediate is mediated by the conserved SMN complex, whereas vertebrate-specific phosphorylation of pICln adds a regulatory dimension that can facilitate or accelerate 6S ring opening under conditions of ULK1 activity, rather than serving as a prerequisite for pICln displacement. The AF3-predicted interface offers a plausible explanation for how C-terminal phosphorylation could modulate the pICln/SmG interaction, but it will require further experimental validation. Future work could include high-resolution cryo-EM of the full-length human 6S complex to visualise the C-terminal segment *in situ*, and targeted mutagenesis of the predicted pICln/SmG contact residues combined with quantitative binding assays. These approaches would enable direct testing of the AF3-derived model and clarify the structural impact of vertebrate-specific phosphorylation.

## CRediT authorship contribution statement

**Christoph Peter:** Writing – review & editing, Writing – original draft, Validation, Supervision, Resources, Formal analysis, Conceptualization. **Sebastian Alers:** Writing – review & editing, Writing – original draft, Conceptualization. **Marco Reinhart:** Visualization, Software, Resources, Formal analysis. **Matthias Grimmler:** Writing – review & editing, Writing – original draft, Formal analysis, Conceptualization.

## Declaration of Generative AI and AI-assisted technologies in the writing process

During the preparation of this work the authors used ChatGPT and DeepL to improve language clarity, consistency, and readability. After using these tools, the authors reviewed and edited the content as needed and take full responsibility for the content of the publication.

## Funding

Not applicable.

## Declaration of Competing Interest

M.G. is employed at DiaServe Laboratories GmbH (Iffeldorf, Germany). S.A. is employed by DiaSys Diagnostic Systems GmbH (Holzheim, Germany). M.R. was employed at GfA GmbH (Pfronten, Germany) during the decisive phases of this project. The remaining author declares that the research was conducted in the absence of any commercial or financial relationships that could be construed as a potential conflict of interest.

## Data Availability

The data underlying this article are available in the article and in its online supplementary material.

## References

[1] Matera A.G., Wang Z. (2014). A day in the life of the spliceosome. Nat Rev Mol Cell Biol.

[2] Maniatis T., Reed R. (1987). The role of small nuclear ribonucleoprotein particles in pre-mRNA splicing. Nature.

[3] Kambach C., Walke S., Young R., Avis J.M., de la Fortelle E., Raker V.A. (1999). Crystal structures of two Sm protein complexes and their implications for the assembly of the spliceosomal snRNPs. Cell.

[4] Raker V.A., Plessel G., Luhrmann R. (1996). The snRNP core assembly pathway: identification of stable core protein heteromeric complexes and an snRNP subcore particle in vitro. EMBO J.

[5] Meister G., Fischer U. (2002). Assisted RNP assembly: SMN and PRMT5 complexes cooperate in the formation of spliceosomal UsnRNPs. EMBO J.

[6] Raker V.A., Hartmuth K., Kastner B., Luhrmann R. (1999). Spliceosomal U snRNP core assembly: Sm proteins assemble onto an Sm site RNA nonanucleotide in a specific and thermodynamically stable manner. Mol Cell Biol.

[7] Meister G., Buhler D., Pillai R., Lottspeich F., Fischer U. (2001). A multiprotein complex mediates the ATP-dependent assembly of spliceosomal U snRNPs. Nat Cell Biol.

[8] Meister G., Eggert C., Buhler D., Brahms H., Kambach C., Fischer U. (2001). Methylation of Sm proteins by a complex containing PRMT5 and the putative U snRNP assembly factor pICln. Curr Biol.

[9] Chari A., Golas M.M., Klingenhager M., Neuenkirchen N., Sander B., Englbrecht C. (2008). An assembly chaperone collaborates with the SMN complex to generate spliceosomal SnRNPs. Cell.

[10] Grimm C., Chari A., Pelz J.P., Kuper J., Kisker C., Diederichs K. (2013). Structural basis of assembly chaperone- mediated snRNP formation. Mol Cell.

[11] Pu W.T., Krapivinsky G.B., Krapivinsky L., Clapham D.E. (1999). pICln inhibits snRNP biogenesis by binding core spliceosomal proteins. Mol Cell Biol.

[12] Barbarossa A., Antoine E., Neel H., Gostan T., Soret J., Bordonne R. (2014). Characterization and in vivo functional analysis of the Schizosaccharomyces pombe ICLN gene. Mol Cell Biol.

[13] Massenet S., Pellizzoni L., Paushkin S., Mattaj I.W., Dreyfuss G. (2002). The SMN complex is associated with snRNPs throughout their cytoplasmic assembly pathway. Mol Cell Biol.

[14] Pellizzoni L., Yong J., Dreyfuss G. (2002). Essential role for the SMN complex in the specificity of snRNP assembly. Science.

[15] Yong J., Kasim M., Bachorik J.L., Wan L., Dreyfuss G. (2010). Gemin5 delivers snRNA precursors to the SMN complex for snRNP biogenesis. Mol Cell.

[16] Hu Y., Hou Y., Zhou S., Wang Y., Shen C., Mu L. (2023). Mechanism of assembly of snRNP cores assisted by ICln and the SMN complex in fission yeast. iScience.

[17] Kroiss M., Schultz J., Wiesner J., Chari A., Sickmann A., Fischer U. (2008). Evolution of an RNP assembly system: a minimal SMN complex facilitates formation of UsnRNPs in Drosophila melanogaster. Proc Natl Acad Sci USA.

[18] Schmitz K., Cox J., Esser L.M., Voss M., Sander K., Loffler A. (2021). An essential role of the autophagy activating kinase ULK1 in snRNP biogenesis. Nucleic Acids Res.

[19] Esser L.M., Schmitz K., Hillebrand F., Erkelenz S., Schaal H., Stork B. (2023). Phosphorylation of pICln by the autophagy activating kinase ULK1 regulates snRNP biogenesis and splice activity of the cell. Comput Struct Biotechnol J.

[20] Jumper J., Evans R., Pritzel A., Green T., Figurnov M., Ronneberger O. (2021). Highly accurate protein structure prediction with AlphaFold. Nature.

[21] Bryant P., Pozzati G., Elofsson A. (2022). Improved prediction of protein-protein interactions using AlphaFold2. Nat Commun.

[22] Abramson J., Adler J., Dunger J., Evans R., Green T., Pritzel A. (2024). Accurate structure prediction of biomolecular interactions with AlphaFold 3. Nature.

[23] Jubb H.C., Higueruelo A.P., Ochoa-Montano B., Pitt W.R., Ascher D.B., Blundell T.L. (2017). Arpeggio: A Web Server for Calculating and Visualising Interatomic Interactions in Protein Structures. J Mol Biol.

[24] Capra J.A., Singh M. (2007). Predicting functionally important residues from sequence conservation. Bioinformatics.

[25] Schedlbauer A., Gandini R., Kontaxis G., Paulmichl M., Furst J., Konrat R. (2011). The C-terminus of ICln is natively disordered but displays local structural preformation. Cell Physiol Biochem.

[26] Yi H., Mu L., Shen C., Kong X., Wang Y., Hou Y. (2020). Negative cooperativity between Gemin2 and RNA provides insights into RNA selection and the SMN complex's release in snRNP assembly. Nucleic Acids Res.

[27] Kliche J., Ivarsson Y. (2022). Orchestrating serine/threonine phosphorylation and elucidating downstream effects by short linear motifs. Biochem J.

[28] Kussie P.H., Gorina S., Marechal V., Elenbaas B., Moreau J., Levine A.J. (1996). Structure of the MDM2 oncoprotein bound to the p53 tumor suppressor transactivation domain. Science.

[29] Craig A.L., Burch L., Vojtesek B., Mikutowska J., Thompson A., Hupp T.R. (1999). Novel phosphorylation sites of human tumour suppressor protein p53 at Ser20 and Thr18 that disrupt the binding of mdm2 (mouse double minute 2) protein are modified in human cancers. Biochem J.

[30] Google DeepMind. AlphaFold Server Guides. Available at: 〈https://alphafoldserver.com/guides#section-1:-introducing-alphafold-3〉. Accessed: 12.12.2025.

