# Supplementary material for: Spliceosomal Sm core assembly: AlphaFold 3 predicted structure and phosphorylation-dependent regulation of the human 6S complex
Source: Comput Struct Biotechnol J. 2025 Dec 16;31:51–60. doi: 10.1016/j.csbj.2025.12.013 (PMC12771329; doi:10.1016/j.csbj.2025.12.013)
Supplement: Supplementary file 5 — Supplementary material [file mmc1.pdf]

SUPPLEMENTARY DATA

Spliceosomal Sm Core Assembly: AlphaFold 3 Predicted Structure and Phosphorylation-Dependent Dynamics of the Human 6S Complex

Matthias Grimmler[†], Marco Reinhart[†], Sebastian Alers and Christoph Peter*

† contributed equally to this work
* Corresponding author: Tel. +49 211 81 12196; Email: christoph.peter@uni-duesseldorf.de

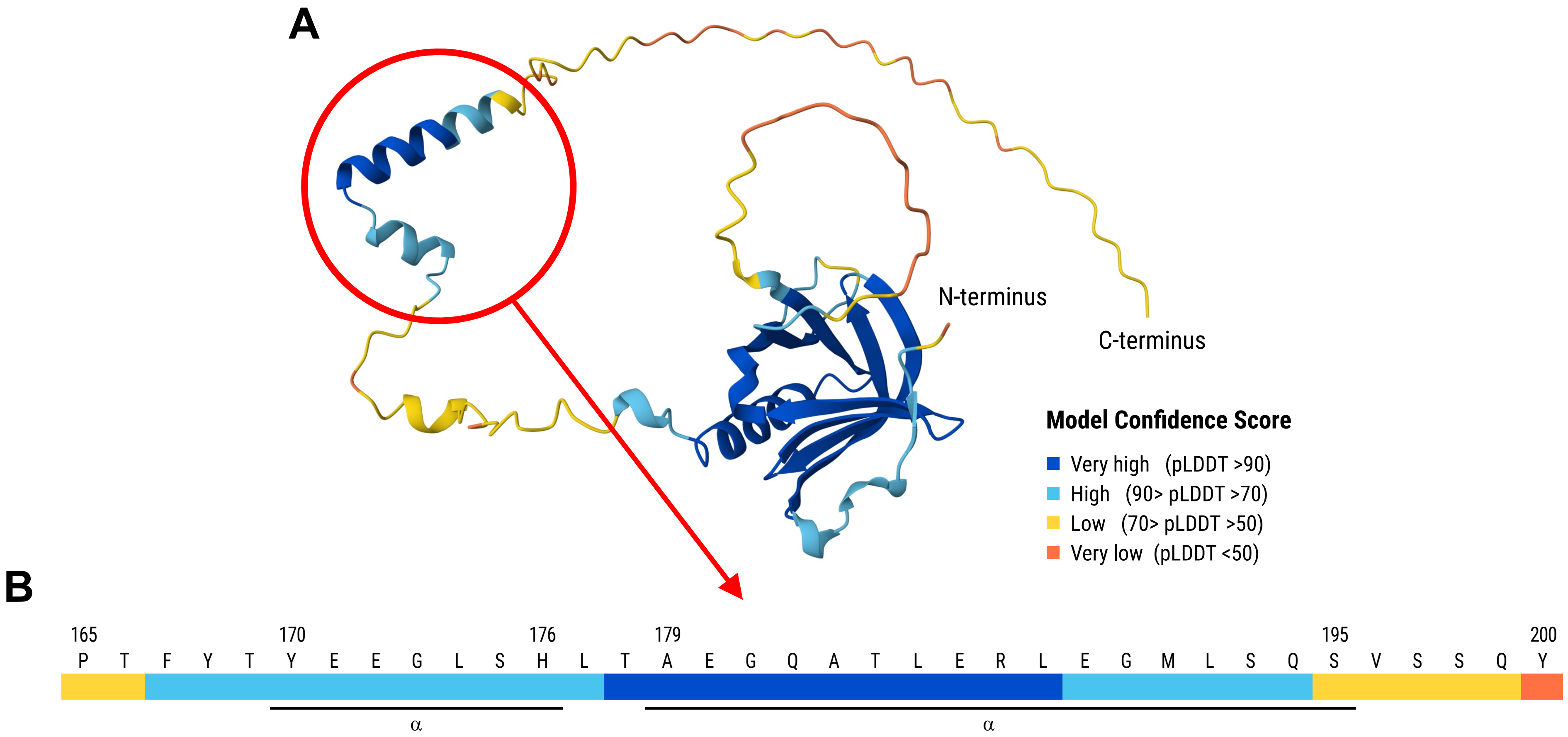

Supplementary Figure S1. Predicted α-helical structure in the mid-C-terminal region of human pICln. **(A)** Structure prediction for human pICln from the AlphaFold Protein Structure Database (Entry AF-P54105-F1-v4) derived from UniProt sequence P54105. Last updated in AlphaFold DB version 2022-11-01, created with the AlphaFold Monomer v2.0 pipeline. Data is available for academic and commercial use, under a CC-BY-4.0 licence. Data available online: https://alphafold.ebi.ac.uk/entry/P54105. **(B)** Schematic representation of the predicted secondary structure and local confidence (pLDDT) score for the isolated pICln monomer between Pro165 and Tyr200.

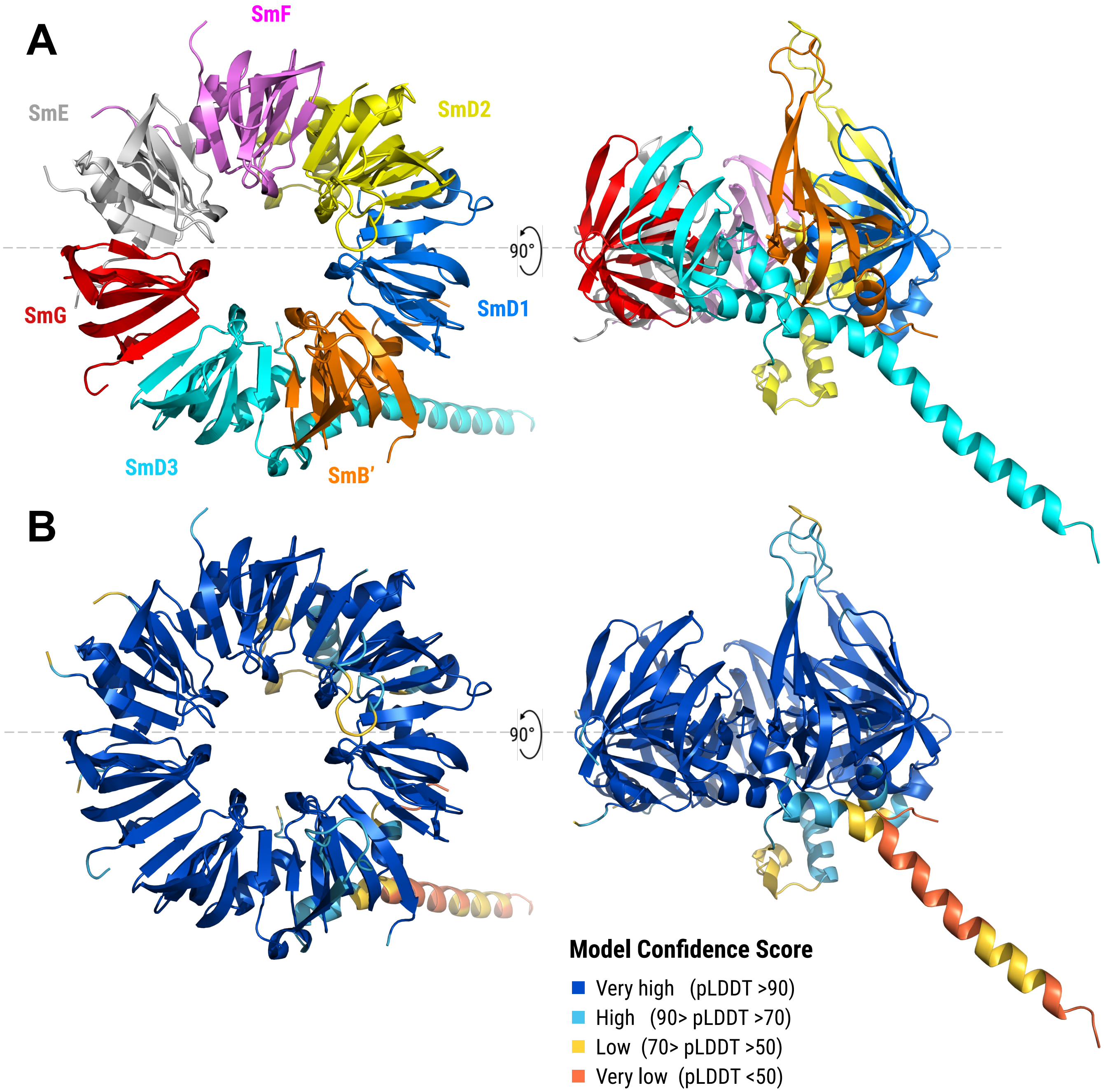

Supplementary Figure S2. Human Sm core predicted by AlphaFold 3. **(A)** Ribbon representation of the hetero-heptameric Sm core comprising the Sm proteins SmB', D1, D2, D3, E, F, and G. The quaternary structure was predicted by AlphaFold 3 using the human reference sequences from UniProtKB. **(B)** Ribbon representation coloured by predicted local distance difference test (pLDDT) score ranging from very low (pLDDT <50) to very high (pLDDT >90).

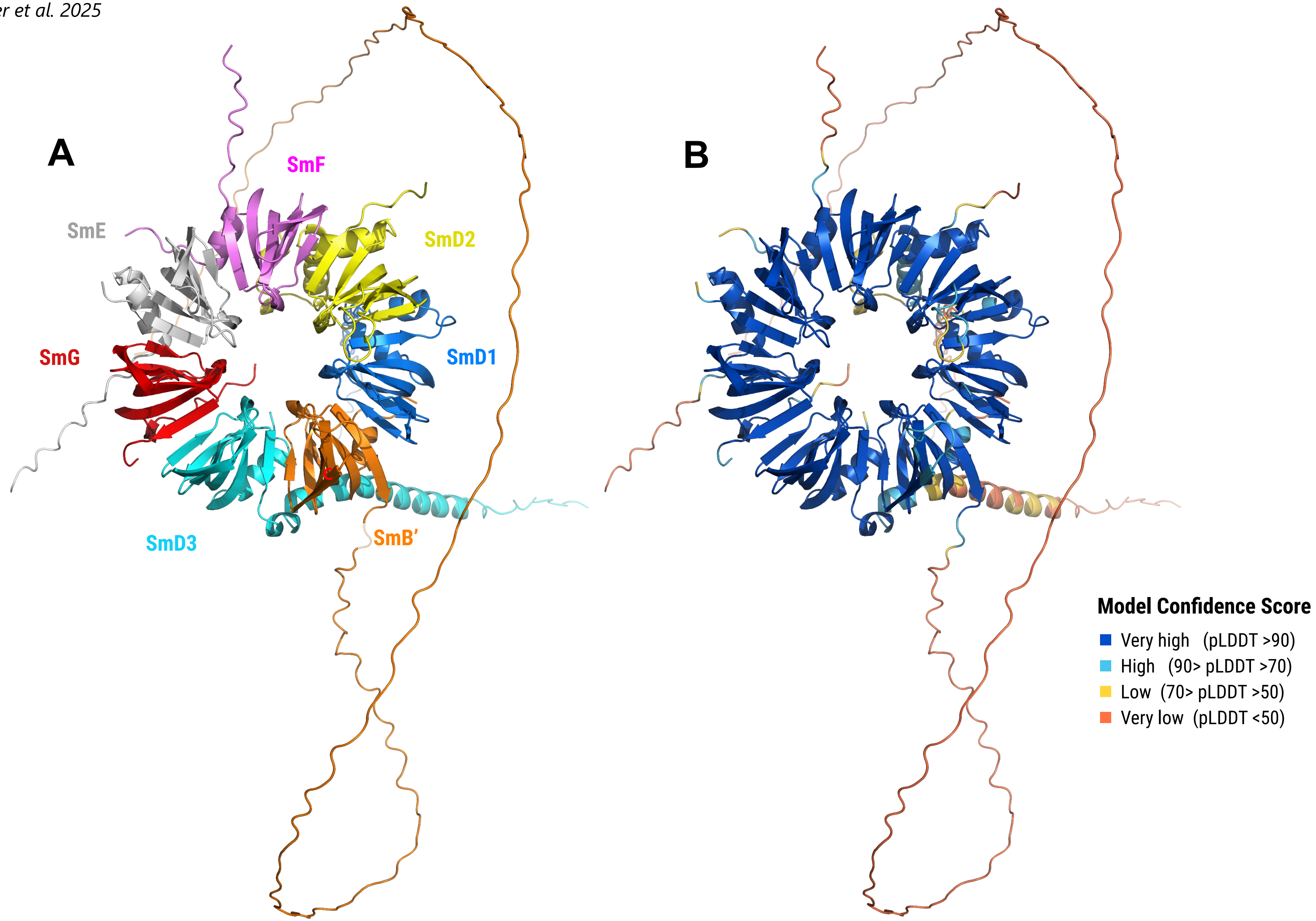

Supplementary Figure S3. Full-length view of the human Sm core predicted by AlphaFold 3.

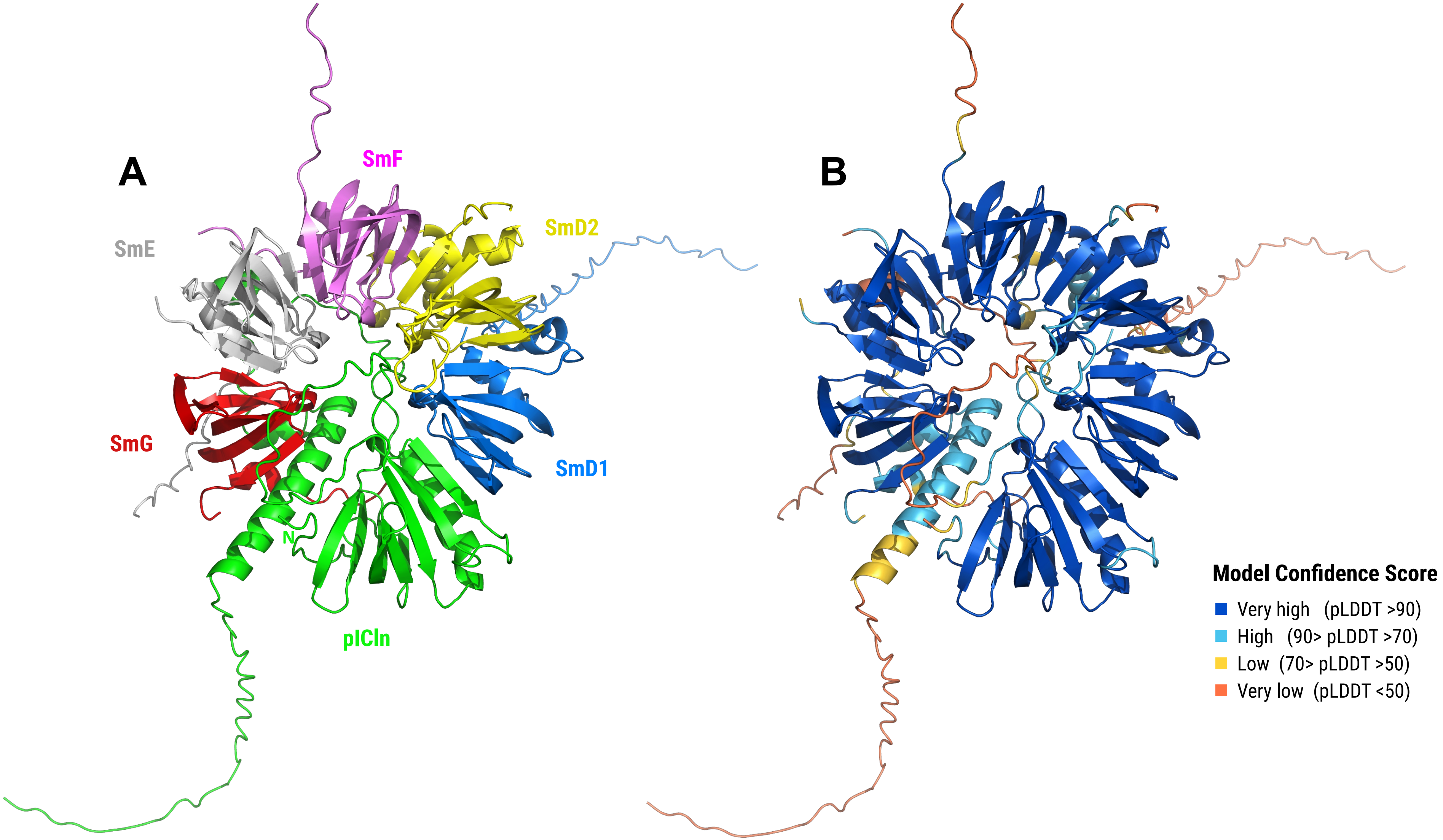

Supplementary Figure S4. Full-length view of the human 6S complex predicted by AlphaFold 3 (Supplement to Figure 1).

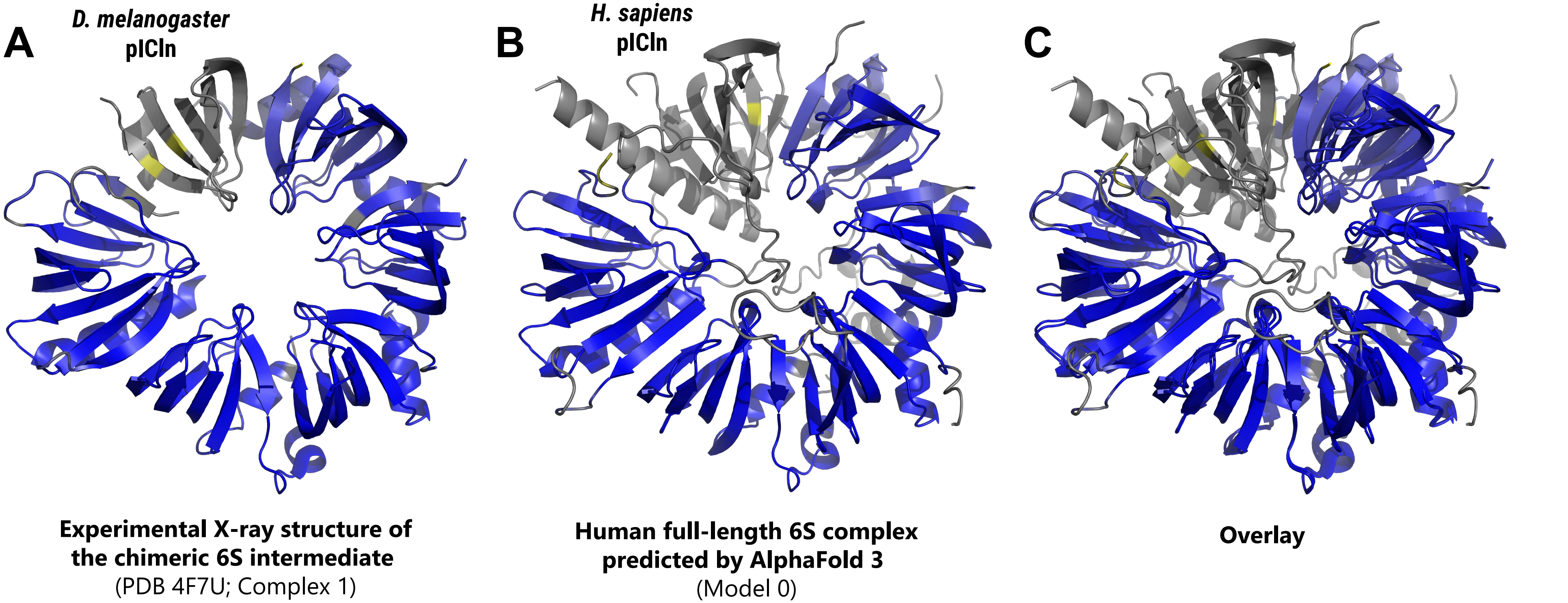

Supplementary Figure S5. Comparison of the experimental X-ray structure of the chimeric 6S intermediate and the AF3-predicted human 6S complex. Positional deviation after superposition in PyMOL are coloured by Root Mean Square Deviation (RMSD) from blue (minimum) to yellow (maximum). **(A)** The X-ray diffraction structure of the chimeric 6S snRNP assembly intermediate (PDB 4F7U), comprising SmD1, D2, E and G from *Mus musculus*, SmF from *Xenopus laevis*, and a truncated pICln from *Drosophila melanogaster* (lacking residues 90–125 and containing an H144A substitution), was superimposed with **(B)** the AF3-predicted 6S complex (Model 0) generated from the full-length human protein sequences. Superposition was performed using the PyMOL *super* command (structure-based, sequence-independent alignment). To improve alignment with the truncated experimental complex, the following unstructured regions were omitted: SmD1 (K86–R119), SmE (M1–Q11), SmF (E76–E86) and pICln (V205–H237). Positional deviation was visualised using a modified ColorByRMSD PyMOL script (https://pymolwiki.org/index.php/ColorByRMSD), adapted to display the Root Mean Square Deviation (RMSD) from blue (minimum) to yellow (maximum). Minimum RMSD: 0.18 Å; Maximum RMSD: 16.41 Å; Average RMSD: 1.64 Å. Structures that could not be aligned are coloured in grey.

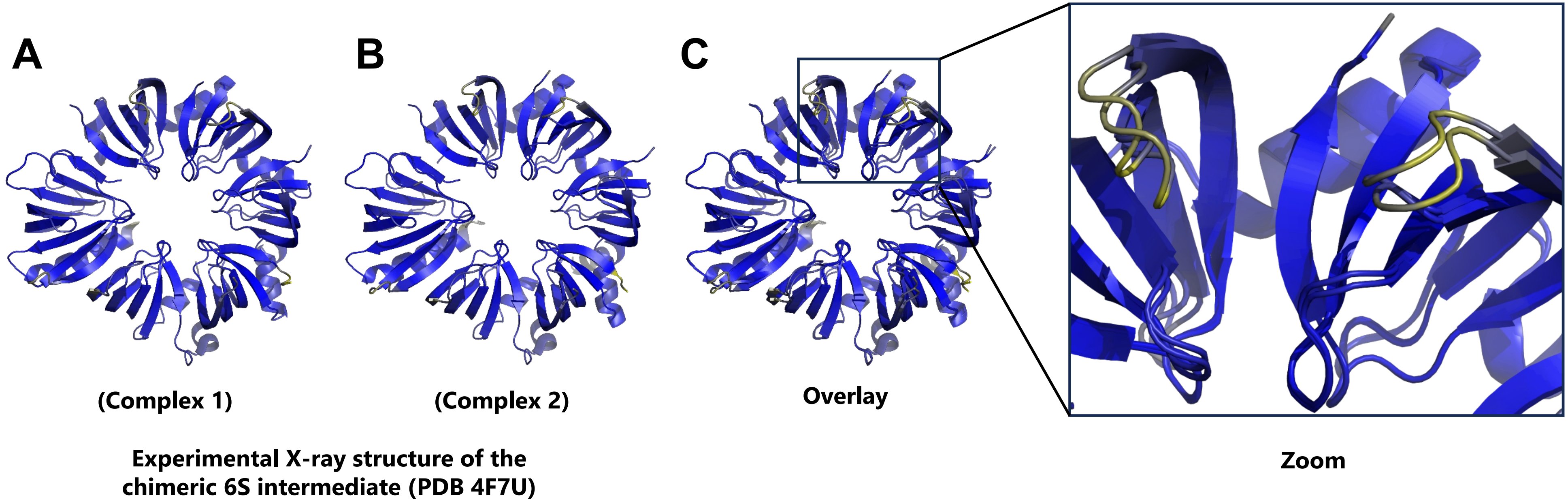

Supplementary Figure S6. Comparison of both chimeric 6S complexes in the asymmetric unit of PDB 4F7U. The experimental crystal structure contains two copies of the 6S complex: **(A)** Complex 1 (chains A, B, E, F, G, P) and **(B)** Complex 2 (chains C, D, H, I, J, Q). **(C)** Both complexes were superimposed using the PyMOL *super* command, and positional deviation was visualised using a modified ColorByRMSD PyMOL script (https://pymolwiki.org/index.php/ColorByRMSD), adapted to display the Root Mean Square Deviation (RMSD) from blue (minimum) to yellow (maximum). Minimum RMSD: 0.06 Å; Maximum RMSD: 3.53 Å; Average RSMD: 0.56 Å. PDB 4F7U chain identity: 4F7U_1 | Chains A, C (SmD1; Mus musculus), 4F7U_2 | Chains B, D (SmD2; Mus musculus), 4F7U_3 | Chains E, H (SmE; Mus musculus), 4F7U_4 | Chains F, I (SmF; Xenopus laevis), 4F7U_5 | Chains G, J (SmG; Mus musculus), 4F7U_6 | Chains P, Q (pICln; Drosophila melanogaster)

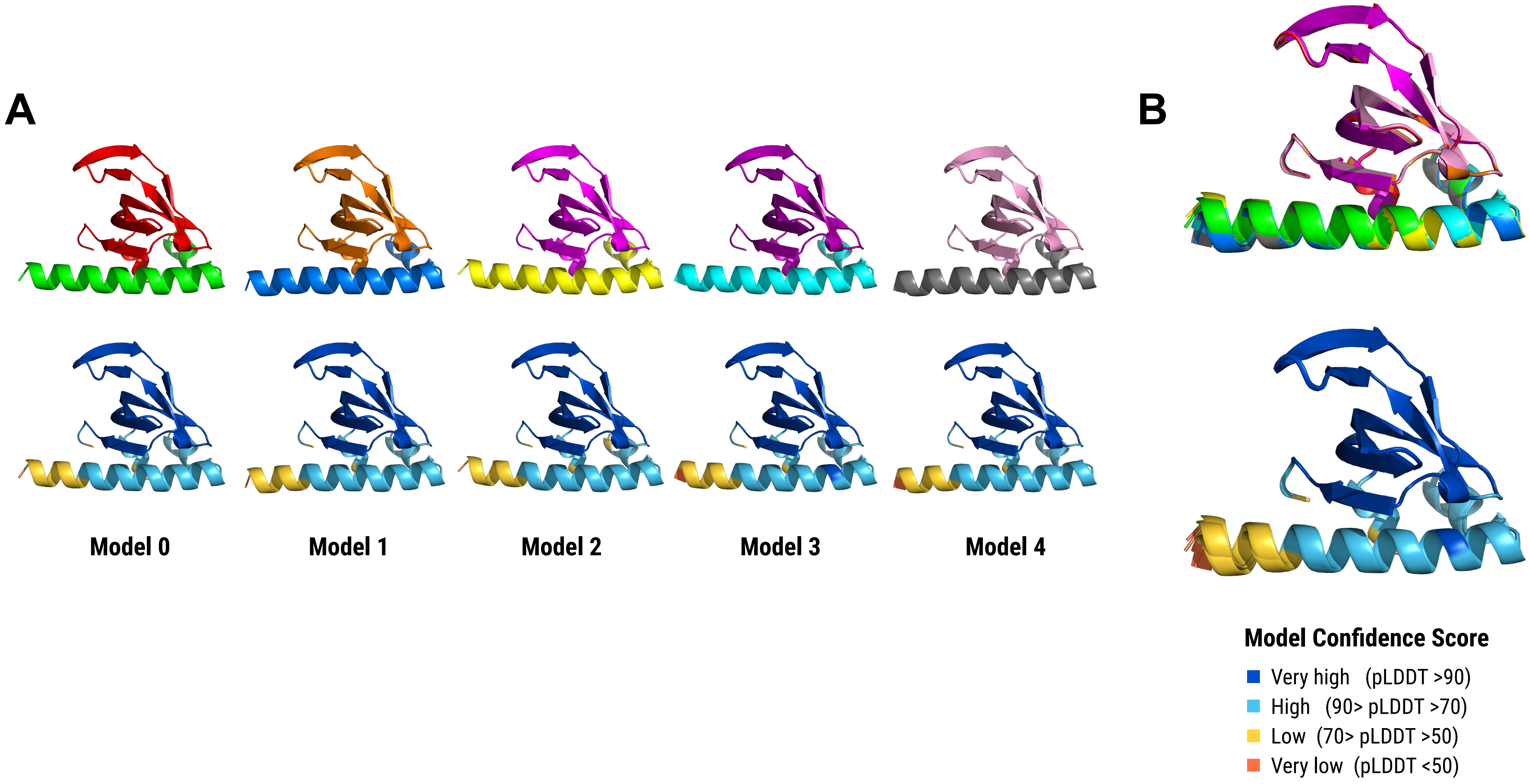

Supplementary Figure S7. The C-terminal α-helix in human pICln and the pICln/SmG contact interface is consistently modelled by AlphaFold 3. **(A)** Comparison of the five ranked AlphaFold 3 models (Models 0–4). For clarity, only SmG (residues E8–V76) and the C-terminal α-helix of pICln (residues T169–G204) are displayed. **(B)** Overlay of all five AF3 models, highlighting the minimal structural variability among the ranked models. The local confidence scores (pLDDT) are depicted in the lower panels.

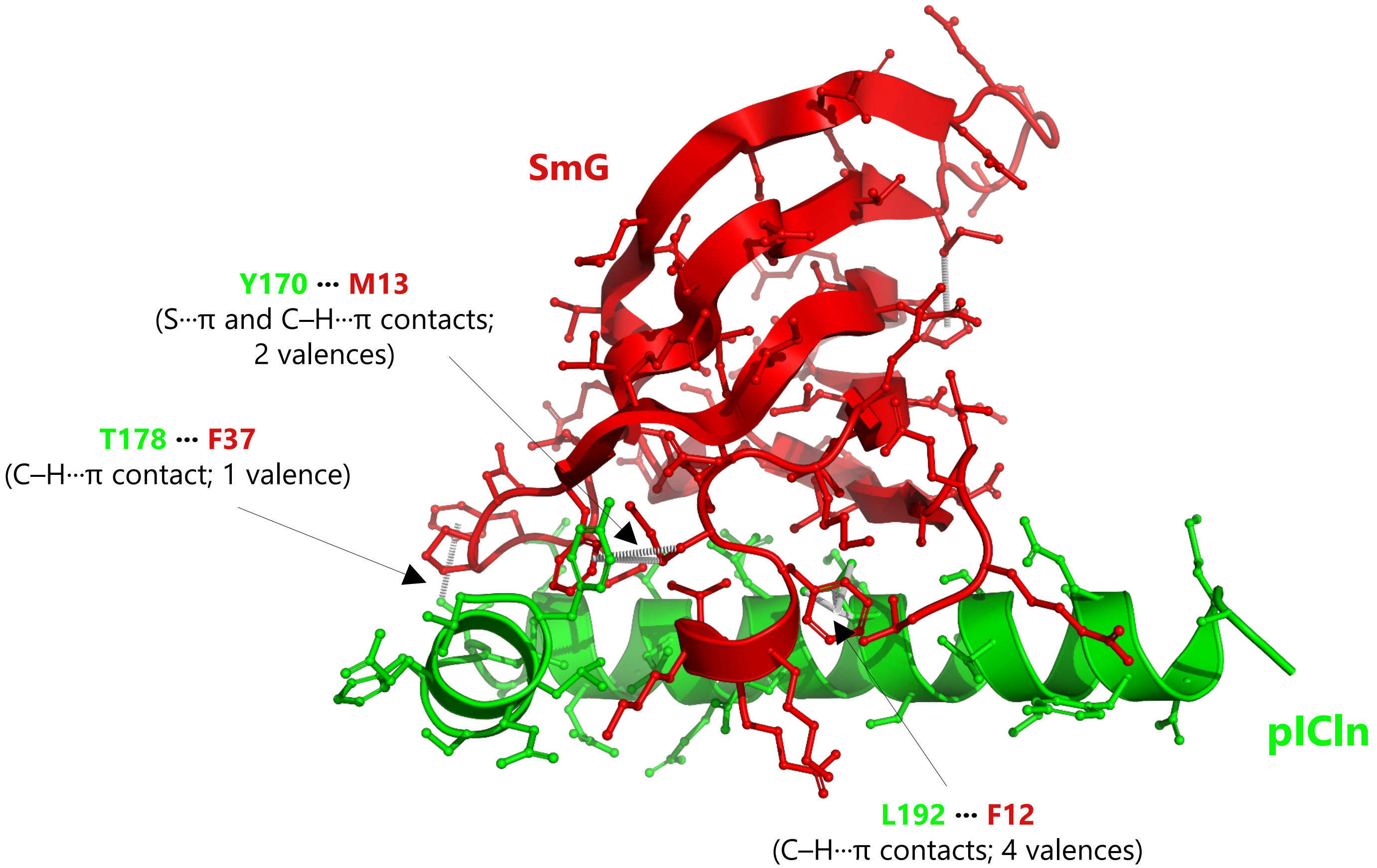

Supplementary Figure S8. Residues in the C-terminal α-helical region of pICln (green) and SmG (red) positioned to mediate additional hydrophobic interactions within the AF3-predicted 6S complex. Side chains geometrically compatible with hydrophobic contacts were identified using the Arpeggio webserver (http://bleoberis.bioc.cam.ac.uk/arpeggioweb; Jubb et al. J Mol Biol. 2017; 429(3):365-371).

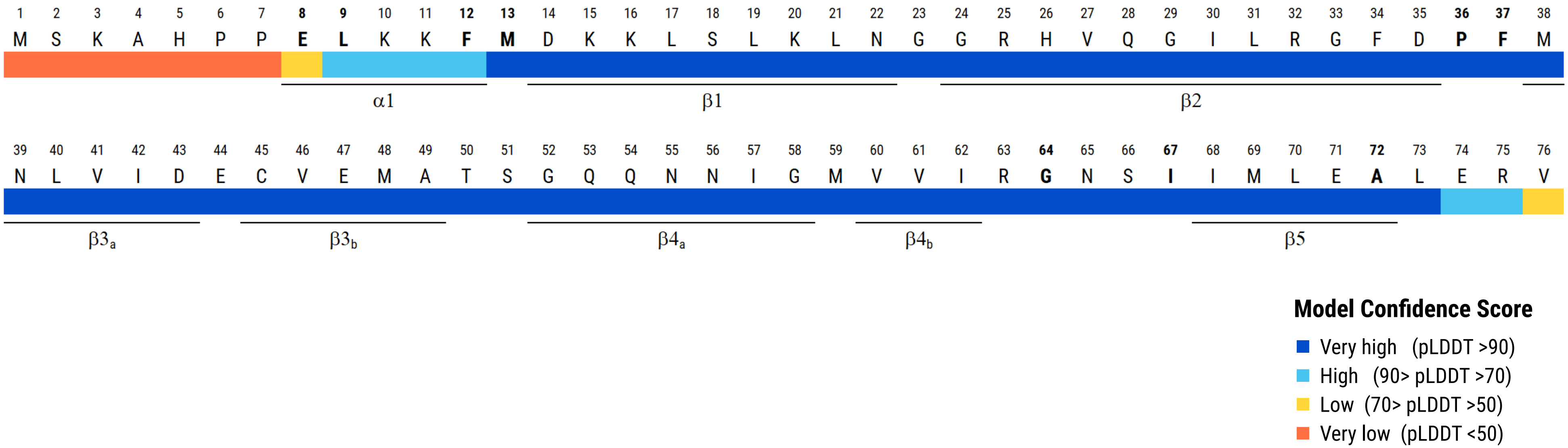

Supplementary Figure S9. (A) Schematic representation of SmG in the human 6S complex predicted by AlphaFold 3. Secondary structure elements (assigned by PyMOL's dss algorithm) and pLDDT scores at each position are indicated. To maintain compatibility with the canonical five-strand annotation (β1-β5) of the Sm fold, the respective highly bent β-strands of SmG were annotated β3a/β3b and β4a/β4b, although the dss algorithm assigns two loops between these β-strands.

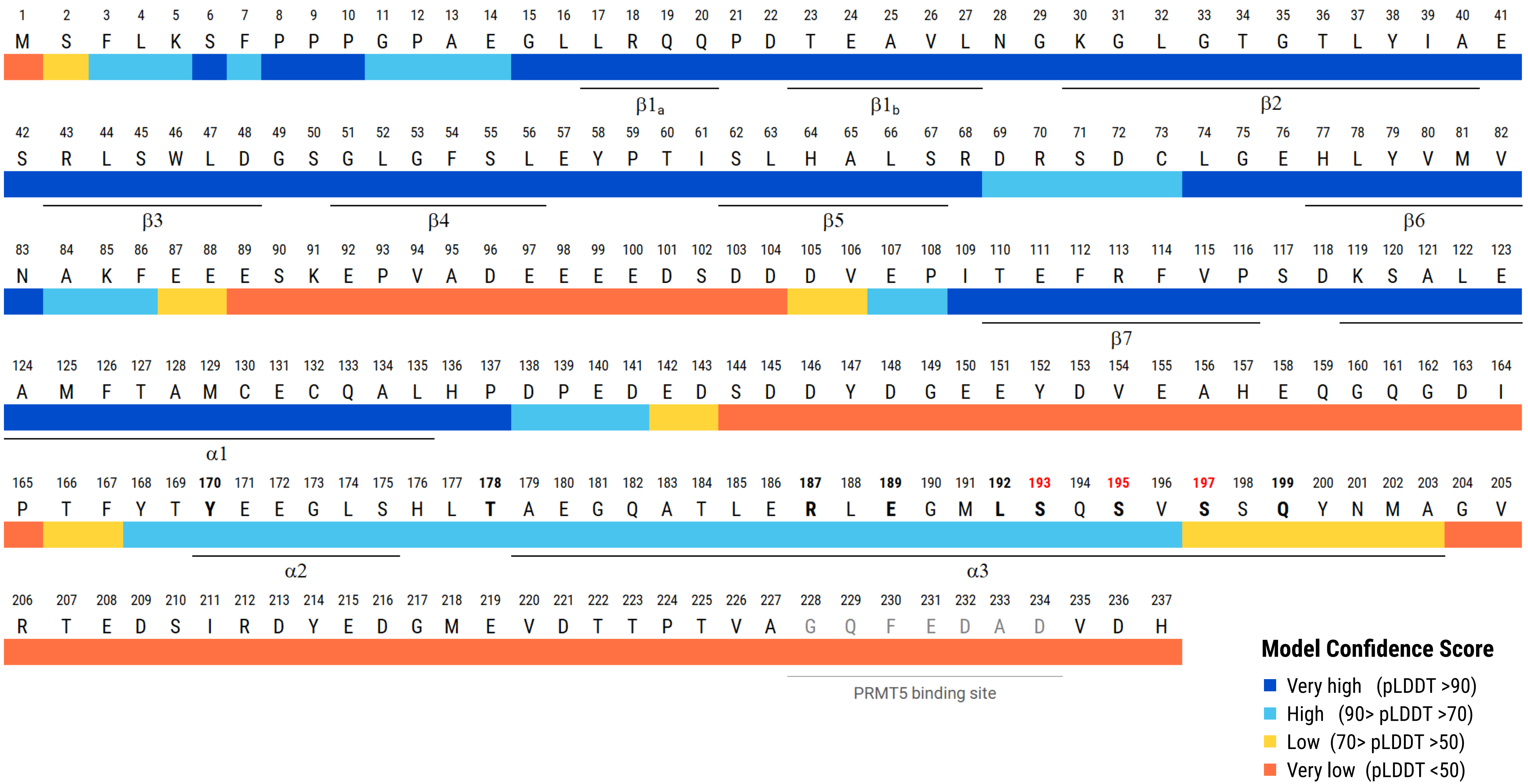

Supplementary Figure S9. (B) Schematic representation of human pICln in the 6S complex predicted by AlphaFold 3. Secondary structure elements (assigned by PyMOL's dss algorithm) and pLDDT scores at each position are indicated. Experimentally determined ULK1-phosphorylation sites in human pICln are highlighted in red, the highly conserved linear PRMT5-binding motif in grey.

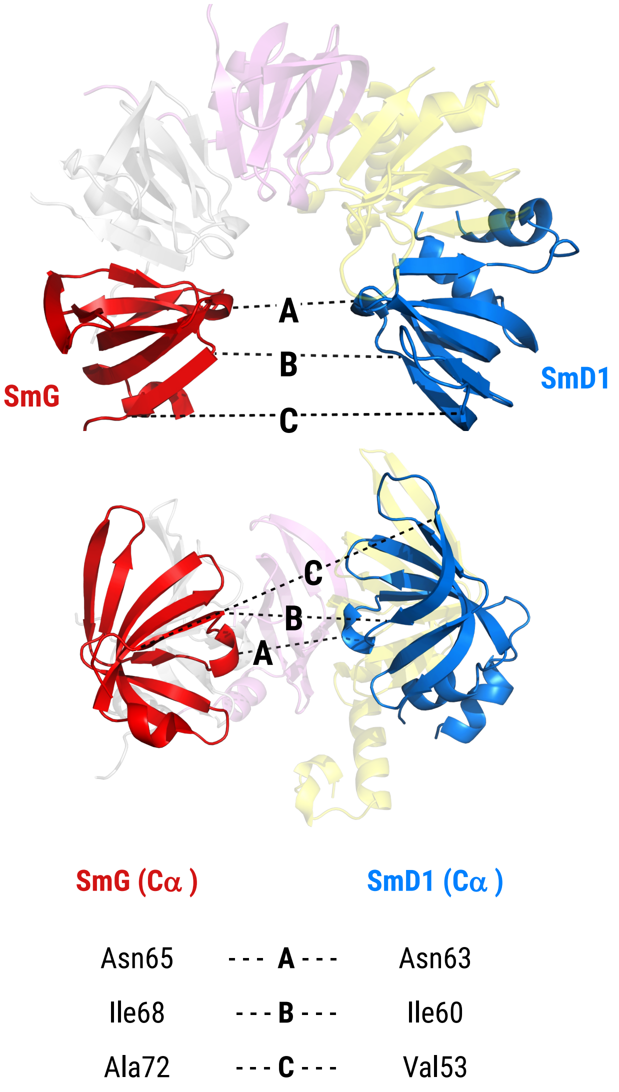

Gap	Complex	Gap distance (Å)						
		Model 0	Model 1	Model 2	Model 3	Model 4	Mean	SD
A	phospho-pICln-6S	15.8	15.9	15.9	16.0	15.7	15.9	
	pICln-6S	15.8	15.8	15.4	15.1	15.9	15.6	
	Gap Δ	0.0	0.1	0.5	0.9	-0.2	**0.3**	**±0.4**
B	phospho-pICln-6S	22.8	23.1	22.8	22.8	22.6	22.8	
	pICln-6S	22.6	22.8	22.1	22.1	22.9	22.5	
	Gap Δ	0.2	0.3	0.7	0.7	-0.3	**0.3**	**±0.4**
C	phospho-pICln-6S	42.6	42.8	42.4	42.5	42.4	42.5	
	pICln-6S	42.4	42.7	41.3	41.5	42.8	42.1	
	Gap Δ	0.2	0.1	1.1	1.0	-0.4	**0.4**	**±0.6**

Supplementary Figure S10. SmG-SmD1 gap distance is unaffected by pICln phosphorylation. The distance between SmG and SmD1 was measured using PyMOL between three identical Cα positions in SmG (red) and SmD1 (blue). Gap A: SmG (Asn65) to SmD1 (Asn63); Gap B: SmG (Ile68) to SmD1 (Ile60); Gap C: SmG (Ala72) to SmD1 (Val53). Gap distances in angstrom (Å) were determined both for the unmodified 6S complex (pICln-6S) and the 6S complex comprising ULK1-phosphorylated pICln (phospho-pICln-6S) for all five AF3 models, and mean gap differences (Gap Δ) and standard deviations (SD) were calculated.

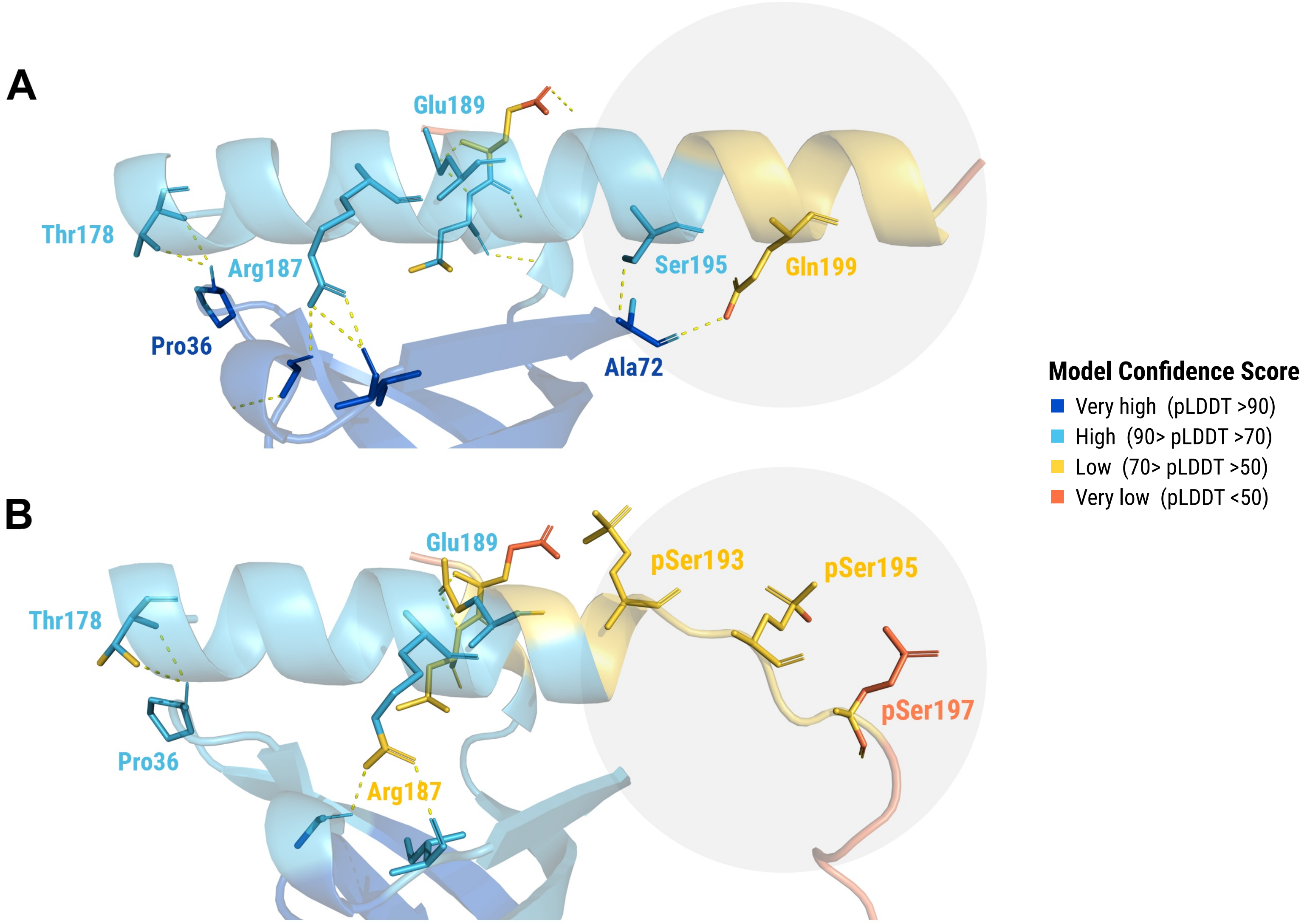

Supplementary Figure S11. The AF3-predicted pICln/SmG binding interface coloured by pLDDT score (Supplement to Figure 4C).

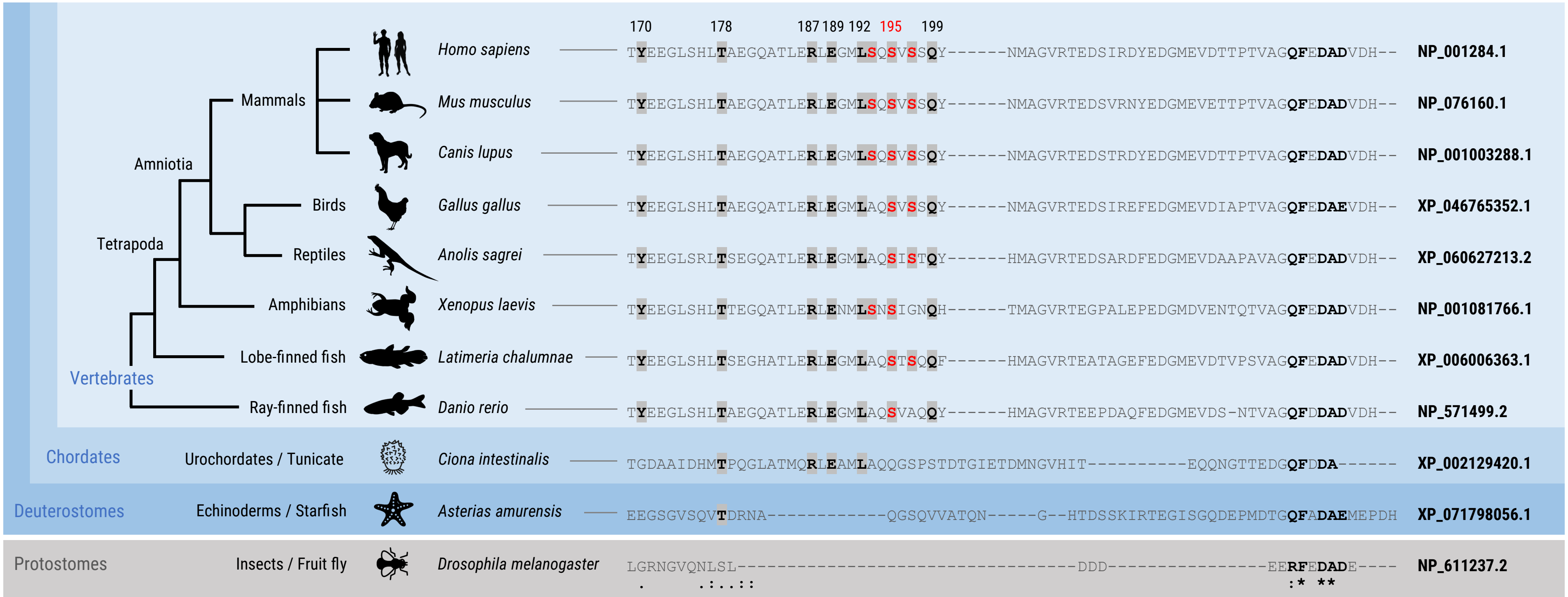

Supplementary Figure S12. The evolutionary conservation of the pICln C-terminus was assessed using Clustal Omega multiple sequence alignment (Version 1.2.4; https://www.ebi.ac.uk/jdispatcher/msa/clustalo). The NCBI Reference Sequence of human pICln (NP_001284.1) was aligned against seven vertebrate homologues ranging from mammals (*Mus musculus, Canis lupus*) to fish (*Latimeria chalumnae, Danio rerio*) using default settings. Evolutionary analysis was extended to three non-vertebrate species: *Ciona intestinalis* (Chordata), *Asterias amurensis* (Deuterostoma) and *Drosophila melanogaster* (Protostoma). Residues conserved across all analysed species (complete identity) are marked with an asterisk (*); conservation of residues with strongly (:) and weakly (.) similar properties according to PAM 250 matrix score are indicated.

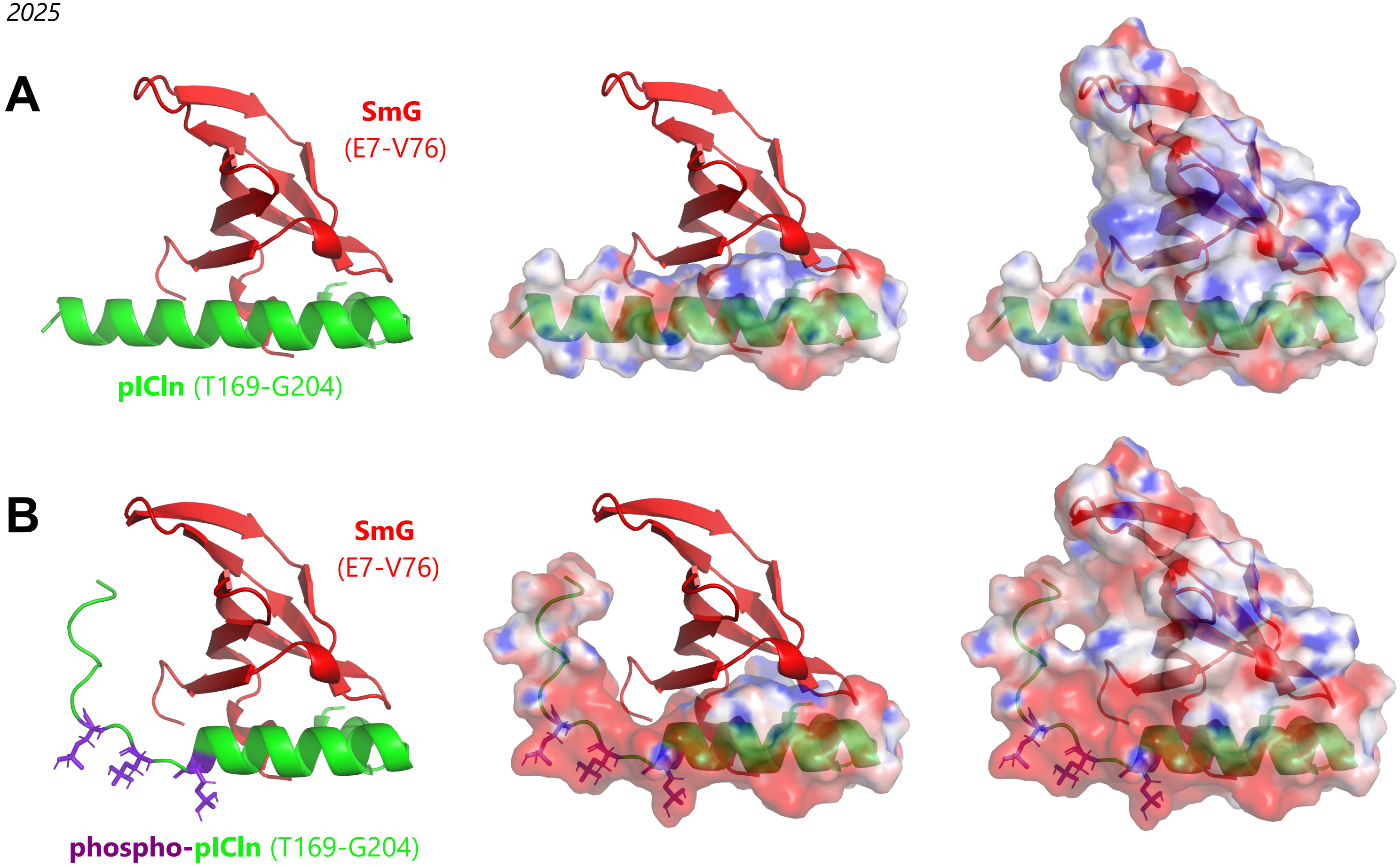

Supplementary Figure S13. Electrostatic surface potential (ESP) at the AF3-modelled pICln/SmG interface coloured from red (−5 kBT/e) to blue (+5 kBT/e). SmG (E7–V76) bound to **(A)** pICln (T169–G204) and **(B)** phospho-pICln (T169–G204) phosphorylated at Ser193, Ser195, Ser197. Note the increased negative potential at the predicted contact region. For ESP mapping, the SmG and pICln fragments were extracted from the atomic coordinate files and saved as separate PDB files. Protonation states/charges for APBS (v1.4) were generated with AmberTools (v24.8; tleap using leaprc.protein.ff14SB and leaprc.phosaa10 to include phosphorylated serine), and written to PQR format with ambpdb. ESPs were computed with APBS (v1.4) to produce DX maps, which were loaded into PyMOL for surface colouring. PDBs were completed with hydrogens at pH 7.0 using PDBFixer (v1.12) to get accurate surface (using surface_mode 1 to include SEP). As these potentials are derived from AF3-predicted coordinates (completed with modelled hydrogens and parameters), the maps should be interpreted qualitatively and treated as hypothesis pending experimental validation.

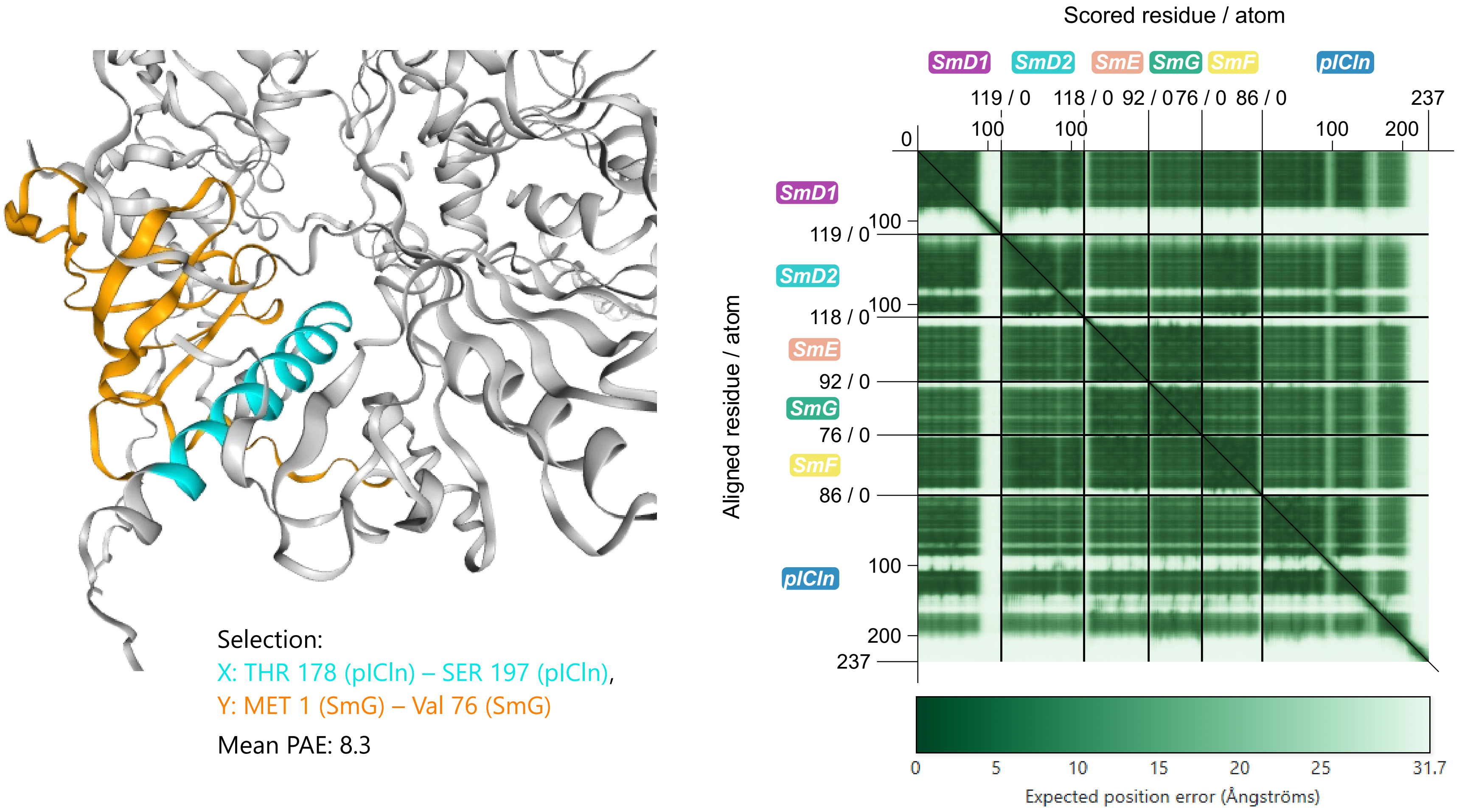

Supplementary Figure S14. Predicted alignment error (PAE) of the AF3-modelled pICln/SmG interface within the human 6S complex. The CIF and JSON files of AlphaFold 3 Models 0–4 were analysed using the PAE Viewer (https://pae-viewer.uni-goettingen.de; Elfmann C, Stülke J. Nucleic Acids Res. 2023). SmG (Met1–Val76; orange) and the C-terminal helix of pICln (Thr178–Ser197; cyan) were selected in the PAE matrix (right panel), and the mean PAE for this region was computed for each model. Mean PAE: 8.3 ±0.17 Å across all five AF3 models.

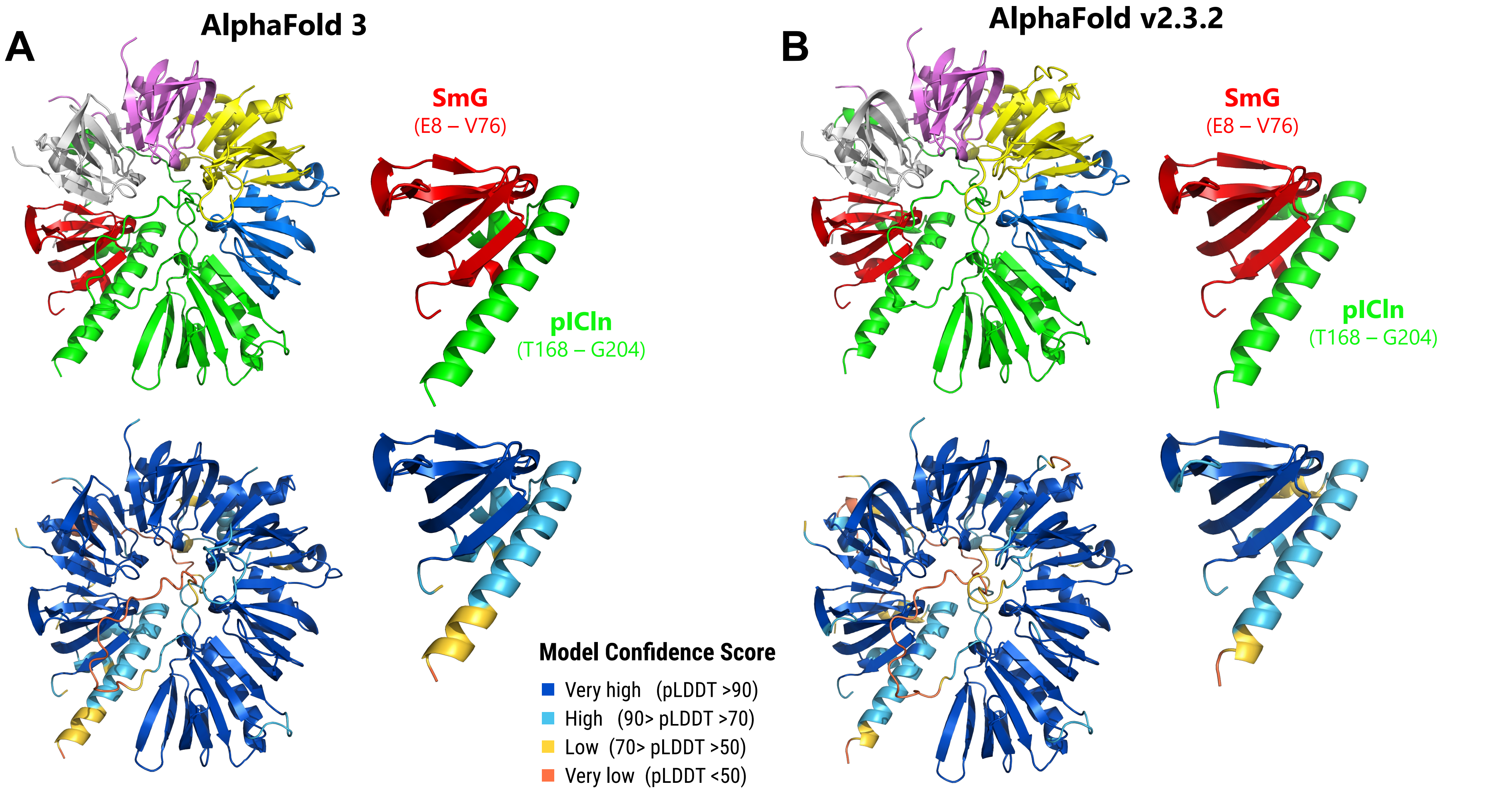

Supplementary Figure S15. Structural comparison between the human 6S complex (Model 0) predicted by **(A)** AlphaFold 3 (https://alphafoldserver.com) accessed between 10 February and 2 March 2025, and **(B)** AlphaFold v2.3.2 (https://github.com/deepmind/alphafold/releases /tag/v2.3.2) run locally on standard hardware (64 GB RAM) using the standard reference databases (Uniprot, UniRef30, UniRef90, MGnify, BFD, PDB70 and PDB). Predictions were performed in CPU-only mode because no CUDA-compatible GPU was available.

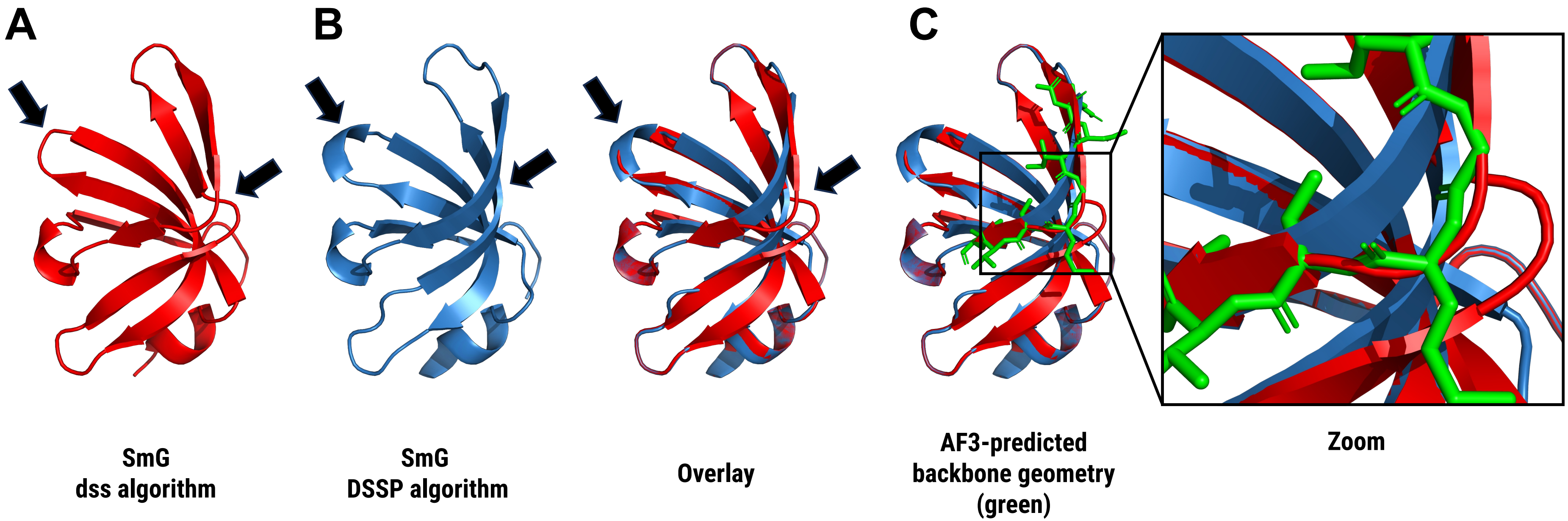

Supplementary Figure S16. Comparison of secondary structure assignments for the AF3-predicted SmG. **(A)** Secondary structure assigned using PyMOL's internal dss algorithm. **(B)** Secondary structure assigned using the DSSP algorithm. **(C)** Overall, the dss-based assignment more accurately reflected the AF3-predicted backbone geometry (green; atomic coordinates in the CIF output file) and was therefore used for all structural representations in this study. To maintain consistency with the canonical five-stranded Sm fold (β1–β5), the highly bent β-strands were annotated as β3a/β3b and β4a/β4b, even though the dss algorithm classifies the intervening regions as loops.

DESCRIPTION OF SUPPLEMENTARY DATA FILES

Supplementary Data 1. AlphaFold 3 output files (Sm core)
Supplementary Data 1 is a ZIP format archive file containing the AlphaFold 3 server output files for all five predicted models (Models 0-4) of the human Sm core, necessary to reproduce the analyses described in this study.

Supplementary Data 2. AlphaFold 3 output files (6S complex)
Supplementary Data 2 is a ZIP format archive file containing the AlphaFold 3 server output files for all five predicted models (Models 0-4) of the human 6S complex.

Supplementary Data 3. AlphaFold 3 output files (phospho-pICln-6S complex)
Supplementary Data 3 is a ZIP format archive file containing the AlphaFold 3 server output files for all five predicted models (Models 0-4) of the human 6S complex, comprising pICln phosphorylated at Ser193, Ser195, and Ser197.

Supplementary Video S1. Structure of the human Sm core predicted by AlphaFold 3
Supplementary Video S1 (mp4) shows a 90° rotation of the human Sm core as depicted in Supplementary Figure S2.

Supplementary Video S2. Structure of the human 6S complex predicted by AlphaFold 3
Supplementary Video S2 (mp4) shows a 90° rotation of the human 6S complex as depicted in Figure 1.

Supplementary Video S3. Structure of the pICln-SmG interface predicted by AlphaFold 3
Supplementary Video S3 (mp4) shows a two-step rotation of the pICln-SmG binding interface within the human 6S complex as depicted in Figure 3A and Figure 4C.

Supplementary Video S4. Structure of the phospho-pICln-SmG interface predicted by AlphaFold 3
Supplementary Video S4 (mp4) shows a two-step rotation of the pICln-SmG binding interface, when pICln is phosphorylated at Ser193, Ser195, and Ser197, as depicted in Figure 4C.
